# Louse-borne relapsing fever—A systematic review and analysis of the literature: Part 1—Epidemiology and diagnostic aspects

**DOI:** 10.1371/journal.pntd.0008564

**Published:** 2021-03-11

**Authors:** Pascal Kahlig, Daniel H. Paris, Andreas Neumayr

**Affiliations:** 1 Swiss Tropical and Public Health Institute, Basel, Switzerland; 2 University of Basel, Basel, Switzerland; 3 Department of Public Health and Tropical Medicine, College of Public Health, Medical and Veterinary Sciences, James Cook University, Queensland, Australia; UAMS, UNITED STATES

## Abstract

Louse-borne relapsing fever (LBRF) is a classical epidemic disease, which in the past was associated with war, famine, poverty, forced migration, and crowding under poor hygienic conditions around the world. The disease’s causative pathogen, the spirochete bacterium *Borrelia recurrentis*, is confined to humans and transmitted by a single vector, the human body louse *Pediculus humanus*. Since the disease has had its heyday before the days of modern medicine, many of its aspects have never been formally studied and to date, remain incompletely understood. In order to shed light on some of these aspects, we have systematically reviewed the accessible literature on LBRF, since the recognition of its mode of transmission in 1907, and summarized the existing data on epidemiology and diagnostic aspects of the disease. Publications were identified by using a predefined search strategy on electronic databases and a subsequent review of the reference lists of the obtained publications. All publications reporting patients with a confirmed diagnosis of LBRF published in English, French, German, and Spanish since 1907 were included. Data extraction followed a predefined protocol and included a grading system to judge the certainty of the diagnosis of reported cases. Historically, Ethiopia is considered a stronghold of LBRF. The recognition of LBRF among East African migrants (originating from Somalia, Eritrea, and Ethiopia) arriving to Europe in the course of the recent migration flow from this region suggests that this epidemiological focus ostensibly persists. Currently, there is neither evidence to support or refute active transmission foci of LBRF elsewhere on the African continent, in Latin America, or in Asia. Microscopy remains the most commonly used method to diagnose LBRF. Data are lacking on sensitivity and specificity of most diagnostic methods.

## Author summary

Louse-borne relapsing fever (LBRF) is an ancient epidemic disease, with descriptions dating back to Hippocrates’ times. Linked to war, famine, poverty, forced migration, and crowding under poor hygienic conditions, the disease has accompanied humankind throughout history and, until 100 years ago, the disease was well recognized among physicians in European and North American countries. Since then, the disease has fallen into oblivion, and reports of the disease are largely confined to Ethiopia. However, the disease has recently resurfaced when it was recognized as a cause of fever in East African migrants (originating from Somalia, Eritrea, and Ethiopia) arriving to Europe. Since the disease has had its heyday before the days of modern medicine, many aspects of the disease have never been formally studied and to date, remain incompletely understood. In order to shed light on some of these aspects, we have reviewed and analyzed the accessible literature on LBRF since its mode of transmission was recognized in 1907 and compiled this 2-part review focusing on epidemiology and diagnostic aspects (part 1) and mortality, Jarisch–Herxheimer reaction (JHR), and impact on pregnancy (part 2). We deliberately did not include an analysis on the antimicrobial treatment of LBRF, as Guerrier and Doherty have published a comprehensive meta-analysis on this aspect in 2011.

## Introduction

Louse-borne relapsing fever (LBRF) is an ancient epidemic disease, with descriptions dating back to Hippocrates’ times [1]. Linked to war, famine, poverty, forced migration, and crowding under poor hygienic conditions, the disease has accompanied mankind throughout history and was once even described as the “most epidemic among the epidemic diseases” [2]. The use of the name “relapsing fever” was first documented by Craigie and Henderson during the epidemic which occurred in Edinburgh from 1843 until 1848 [3,4], reviewed by Greig 100 years later [5]. Milestones in the disease’s history were the discovery of the causative organism by Obermeier in Berlin in 1873 [6], the discovery of the organism in the vector by Mackie in India in 1907 [7], and the description of the mode of transmission by Sergent and Foley in Algeria in the same year [8,9]. In history, LBRF had a massive impact, especially following political crisis, socioeconomic disaster, and war [10,11].

Since the disease had its peak incidence and prevalence before the days of modern medicine, many aspects of the disease have never been formally studied and remain incompletely understood to date. In order to shed light on epidemiological and diagnostic aspects, we reviewed and analyzed the available published data on LBRF since its transmission was identified in 1907.

## Epidemiology

With the reduction of the vector *Pediculus humanus*, due to improved living standards along with the introduction of the insecticide dichlorodiphenyltrichloroethane (DDT) in the 1940s, LBRF declined and finally disappeared from most regions of the world, as well as from most medical text books, over the past century [10,12].

**Fig 1:**
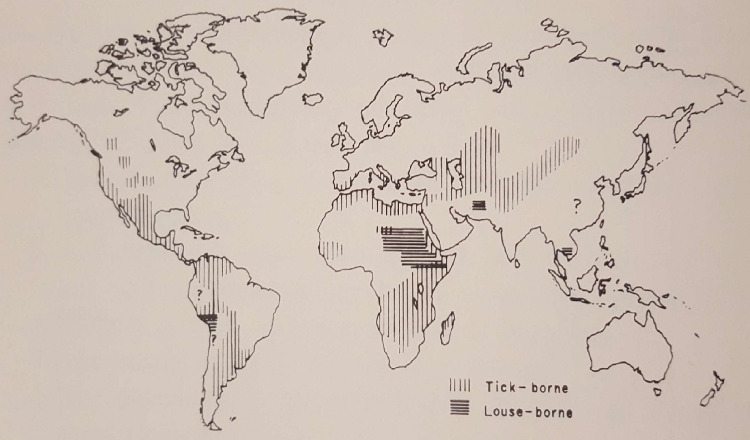
Assumed global distribution of TBRF and LBRF (1950–1969) [25]. LBRF, louse-borne relapsing fever; TBRF, tick-borne relapsing fever.

In the last decades, reports of cases were almost exclusively limited to the Horn of Africa [10,11] and LBRF was increasingly considered a disappearing, neglected tropical disease (NTD) [12] until the disease recently resurfaced as non-malarial febrile illness in East African migrants arriving from Somalia, Eritrea, and Ethiopia to Europe [13–17]. Although several authors extensively reviewed the epidemiology of LBRF in Africa and Europe [18–26] and several studies and book chapters describe remaining endemic foci of LBRF in Africa, South America, and Asia [13,22,24,25,27–36], there is very little reliable data on the disease’s true past and present epidemiology, especially in Latin America and Asia (Fig 1).

In order to shed light on the global epidemiology of LBRF over the past 100 years, we reviewed all available published reports on LBRF cases and summarized their number, their time of occurrence, and the grade of evidence for their correct diagnosis. Additionally, we performed an in-depth analysis of the rather unclear past and present situation of LBRF in Latin America and Asia.

## Diagnostic aspects

**Fig 2:**
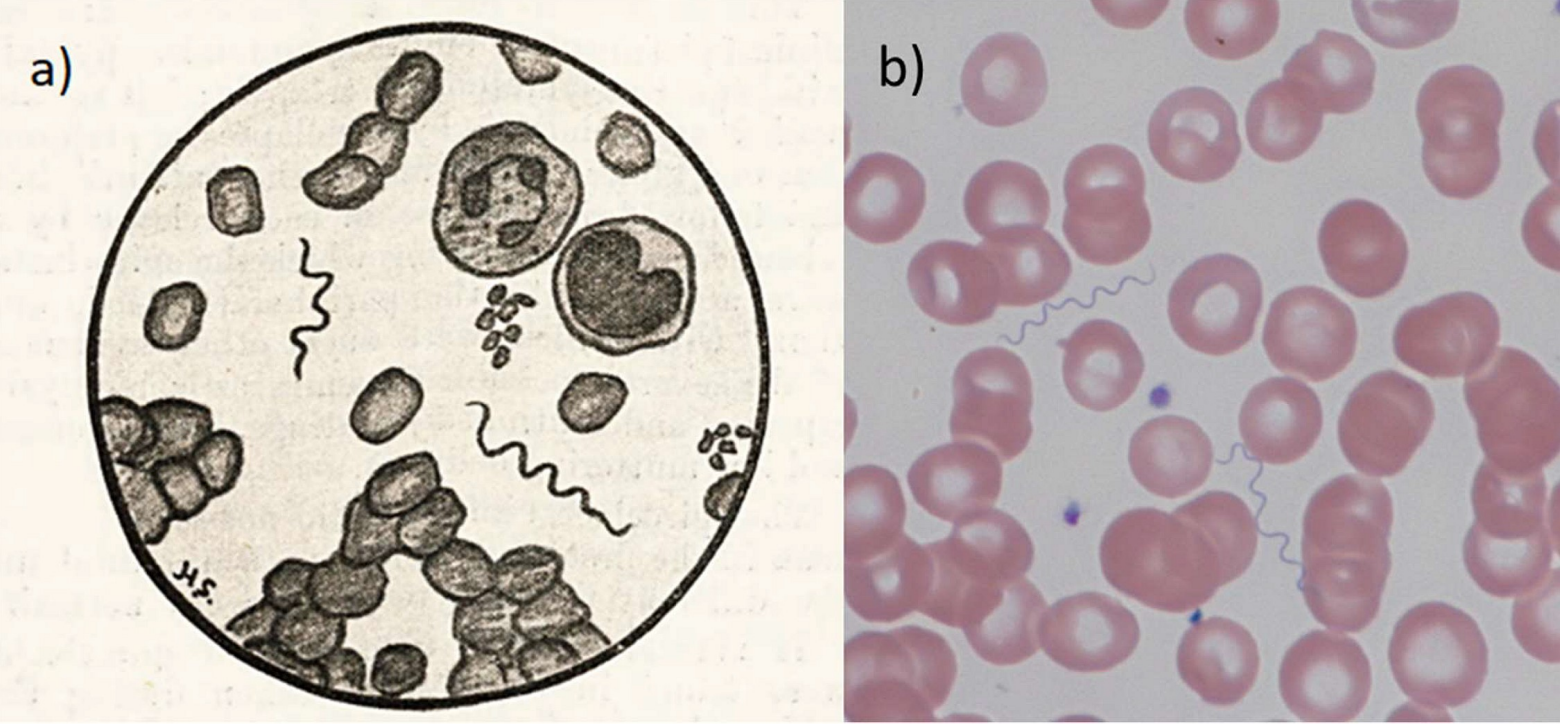
Microscopic detection of *B*. *recurrentis* in blood films. a) Drawing of *B*. *recurrentis* spirochetes found in a thin blood film obtained from a patient suffering from LBRF in 1909 in Jaipur, India [39]. b) Photography of *B*. *recurrentis* spirochetes found in a thin blood smear (May–Grünwald–Giemsa stain, magnification 1,000-fold) obtained from an Eritrean migrant suffering from LBRF in 2015 in Basel, Switzerland. LBRF, louse-borne relapsing fever; TBRF, tick-borne relapsing fever. *Image credit*: *Dr*. *Michael Osthoff*.

Before the causative organism of LBRF was identified in 1873 [6], the diagnosis was exclusively based on signs and symptoms. However, since other febrile illnesses may present with similar signs and symptoms (e.g., “louse-borne typhus” caused by *Rickettsia prowazekii*, typhoid fever, or leptospirosis) as well as recurrent or periodic episodes of fever (e.g., tick-borne relapsing fever [TBRF], malaria, or the louse-borne “trench fever” caused by *Bartonella quintana*), it is probable that these diagnoses were often confused. After the discovery of the causative organism, microscopy of blood films became the diagnostic gold standard for LBRF. In thick and thin blood films (stained with Giemsa, May–Grünwald–Giemsa, Wright, Wright–Giemsa, Field’s, or Diff-Quick stains or examined under dark field), *Borrelia* spirochetes are identifiable by their typical morphology (Fig 2). However, since *Borrelia recurrentis* is microscopically undifferentiatable from *Borrelia* spp. causing TBRF, true diagnostic confirmation of LBRF only became available with the introduction of polymerase chain reaction (PCR) and sequencing techniques in the 1980s. However, considering the disease’s transmission, certain circumstances (e.g., outbreaks, epidemics, and occurrence in a vulnerable population) add conclusive epidemiological evidence and support the microscopical diagnosis as LBRF rather than TBRF. This does not apply to sporadic cases, where the way of transmission (i.e., ticks in an endemic region) rather supports TBRF. Nevertheless, microscopic examination has been the gold standard for diagnosing relapsing fever [37,38].

Serology has been used as alternative diagnostic method, but shows limited specificity due to cross-reactivity among *Borrelia* spp., including TBRF, as well as with other spirochetes (e.g., *Treponema pallidum*) [25,35,36]. Moreover, within endemic regions, the interpretation of serological assays is often complicated by high background reactivity and sero-scars (persistence of detectable antibodies after infection) in addition to the fact that the respective laboratory capacity and expertise are mostly unavailable. Furthermore, since seroconversion demands time, serology is not helpful to diagnose acute infection. Thus, serology was never developed into commercial available assays, and microscopy remains the sole and most widely available diagnostic tool to date [40]. With the introduction of PCR-based methods, the diagnostic sensitivity improved markedly, and species differentiation of relapsing fever *Borrelia* became available [41,42]. However, the availability is still largely restricted to affluent countries and research settings.

Table 1 lists the advantages and disadvantages of the laboratory diagnostic and research methods applied in LBRF.

**Table 1:** Overview on laboratory methods applied in LBRF and their advantages, disadvantages, and use.

Method	Advantage	Disadvantage	Use
**Microscopy**	Fast; widely available	Variability (spirochete density, interobserver variability, and methodological differences); does not allow the differentiation between LBRF and TBRF *Borrelia*	Diagnostic gold standard
**Serology**	Allows retrospective evaluation	Not useful as acute diagnostic method due to delayed seroconversion; does not allow for species differentiation	Epidemiological studies
**PCR**	Species specific; high sensitivity; allows to differentiate LBRF from TBRF Borrelia*	Currently no standardized protocol for discrimination between *Borrelia duttonii* and *B*. *recurrentis*	Largely restricted to research institutions
**Culture**	Isolation and growth of *B*. *recurrentis*	Time and resource demanding, overall challenging	Research only
**Animal inoculation**	Enhanced sensitivity in cases with negative microscopy; differentiation between LBRF and TBRF Borreliae**	Time and resource demanding	Historical research method; also formerly used to “transport” *Borrelia*

LBRF, louse-borne relapsing fever; PCR, polymerase chain reaction; TBRF, tick-borne relapsing fever; WGS, whole genome sequencing.

* Note: With the increasing availability of pan-bacterial 16S rRNA PCR assays as well as WGS technology, the diagnostic repertoire has greatly improved in resource-rich settings in recent years.

** Note: Rodents are susceptible to TBRF *Borreliae*, but refractory to *B*. *recurrentis* infection.

In order to shed light on the evolution of LBRF diagnostics over the past 100 years and the accuracy of different test methods, we reviewed the available literature and summarized the available data.

## Methods

A systematic review protocol established for this review is available in the Supporting information section (S1 Text). The electronic databases BIOSIS, CINAHL, Cochrane Library, Current Contents Connect, Elsevier, EMBASE Ovid, Ovid MEDLINE, PMC, PubMed, Scopus, and Web of Science were searched on October 4, 2017 using the search term ((Relapsing AND fever AND (Louse OR Lice OR (Pediculus AND humanus))) OR (Borrelia AND recurrentis) OR LBRF). A second and third search, using the same search term on the same databases, was conducted on August 7, 2018 and June 17, 2019, respectively. After checking for and removing duplicates (using EndNote software and manually [43]), publications were prescreened by checking titles and abstracts if they concerned patient(s) with the diagnosis of LBRF. Publications not reporting patient(s) with the diagnosis of LBRF were excluded. The remaining publications were then full-text assessed for fulfillment of the inclusion criteria: reporting conclusively diagnosed case(s) of LBRF and published after 1907 (the year when the disease’s mode of transmission was discovered) and published in English, French, German, or Spanish. Publications not fulfilling the inclusion criteria were excluded. Publications that could neither be retrieved through their respective journals, nor by contacting libraries, or after contacting the authors, were classified as “not retrievable” and excluded. Additional relevant publications identified when reading the full-text articles or checking their reference lists were reviewed and included if they fulfilled the inclusion criteria (“snowball” search strategy).

A data extraction sheet for screening and selecting eligible publications was developed and is available in the Supporting information section (S2 Text). The following data were extracted from eligible publications using a standardized excel spreadsheet: patient characteristics (number of patients, age, gender, origin, occupation, social status, and way and duration of migration), diagnostic method (microscopy and molecular method), symptoms and signs (fever, chills, myalgia, headache, hepatomegaly, splenomegaly, signs of hemorrhage, and others), treatment (number of treated and untreated patients, drug, dosage, and duration and route of administration), and outcome (Jarisch–Herxheimer reaction (JHR), abortion/stillbirth, premature delivery, and mortality).

To minimize bias, the same reviewer conducted a second full data extraction more than 1 month after the first extraction. Discrepant results and unclear cases were resolved by consulting a second reviewer.

In order to consider the probability of a correct diagnosis of LBRF, all reviewed cases were graded according to the used diagnostic method and respectively classified (Table 2).

**Table 2:** Diagnostic grading system to judge the certainty of the correct diagnosis of LBRF.

Diagnostic method	Grade of diagnostic certainty	Case classification	Comment
PCR-based method	A	Confirmed diagnosis	Highest level of evidence for correct diagnosis
Microscopy	B	Microscopic diagnosis	Second highest level of evidence for correct diagnosis; microscopic identification of spirochetes during LBRF epidemics or in countries with current endemic foci leaves little doubt of the certainty of the diagnosis and may be regarded with an almost equal level of certainty as grade A
Paired serology	C	Indirect evidence	Intermediate level of evidence for correct diagnosis due to limited sensitivity and specificity of the method; paired serology, demonstrating seroconversion or increment of titer, is considered superior to single-titer serology
Single-titer serology	D	Indirect evidence	See comment under C above
Clinical diagnosis	E	Clinical diagnosis	Lowest level of evidence for correct diagnosis

LBRF, louse-borne relapsing fever; PCR, polymerase chain reaction.

* Note: Animal inoculation, historically used as supportive diagnostic method in LBRF research, was not considered a means of conclusive diagnosis, and thus not included in the evaluation.

To visualize the worldwide epidemiology of LBRF over the past century, data of all identified cases were entered into a geographic information system (GIS) application (https://www.qgis.org/en/site/) and graphically displayed using geodata from Natural Earth (https://www.naturalearthdata.com/about/terms-of-use/).

The review followed the Preferred Reporting Items for Systematic Reviews and Meta-Analyses (PRISMA) statement (S1 Checklist).

## Results

**Fig 3:**
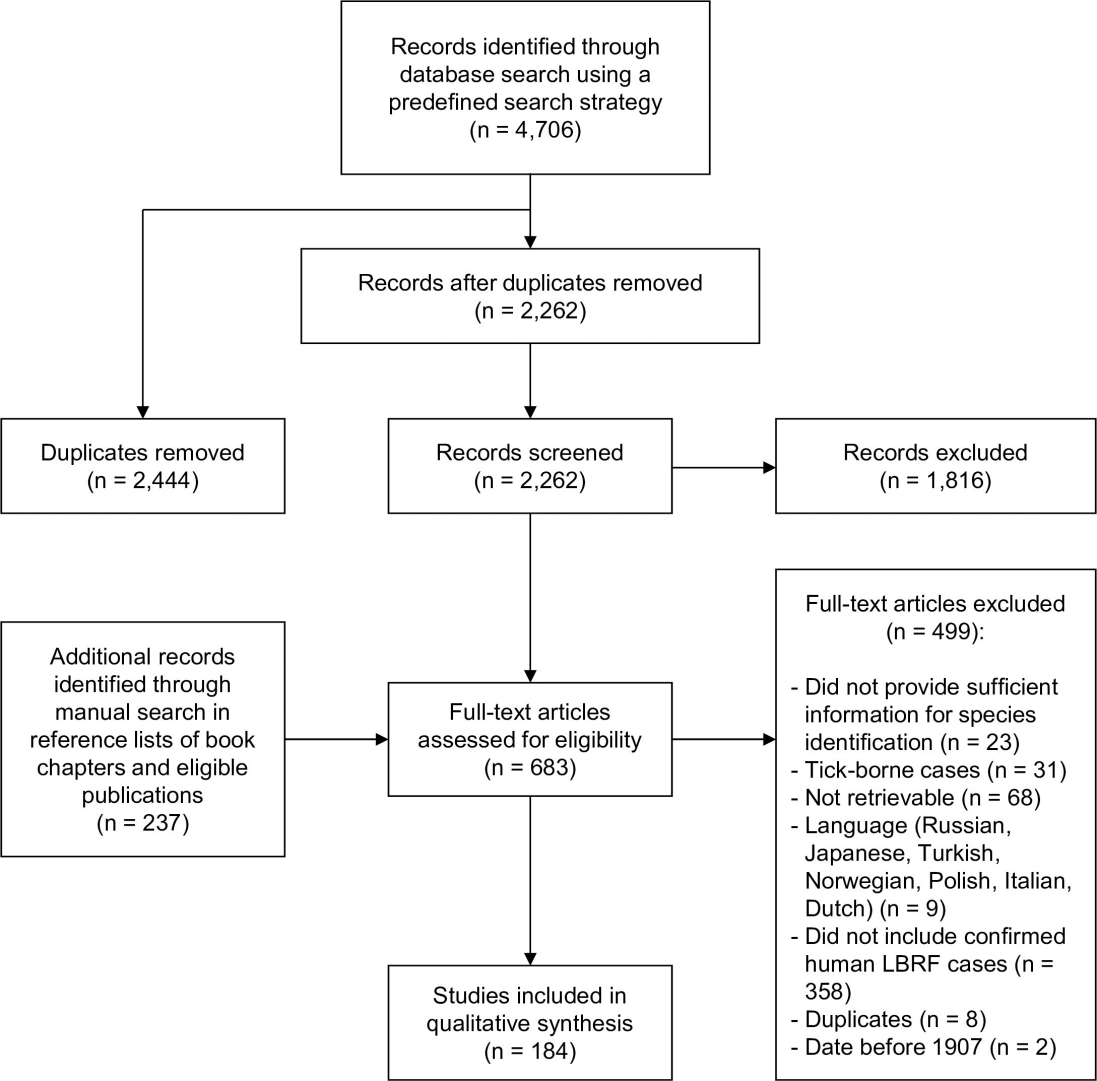
Flow diagram of search and selection of eligible publications. LBRF, louse-borne relapsing fever.

Our search strategy identified 4,943 publications of which 184 finally proved eligible for being included and analyzed (Fig 3, S1 Fig). A list of included and excluded publications is available in the Supporting information section (S3 Text).

From the 184 included publications, data of 18,613 LBRF cases were extracted (S1 Data). A total of 16,632 cases (90%) were microscopically diagnosed, 1,882 cases (10%) were clinically diagnosed, and 99 cases (0.5%) were confirmed by PCR (the majority after primarily being diagnosed by microscopy) (Table 3).

**Table 3:** Number of diagnosed LBRF cases over time according to the diagnostic method used.

Diagnostic method	Grade of diagnostic certainty	Case classification	Reported cases of LBRF diagnosed 1907–2019 *N* (%)											
			1907–1919	1920–1929	1930–1939	1940–1949	1950–1959	1960–1969	1970–1979	1980–1989	1990–1999	2000–2009	2010–2019	1907–2019
PCR-based method	A	Confirmed diagnosis	n.a.	n.a.	n.a.	n.a.	n.a.	n.a.	n.a.	0	24^†^ (0.7)	4 (0.2)	71^‡^ (14)	99
Microscopy	B	Microscopic diagnosis	1,360 (60.1)	2,017 (93.3)	972 (97.8)	4,679 (88.7)	297 (100)	117 (100)	921 (100)	636 (100)	3,183 (97.7)	2,017 (92.4)	433 (85.1)	16,632
Paired serology	C	Indirect evidence	n.a.	n.a.	n.a.	n.a.	n.a.	n.a.	n.a.	n.a.	[33^§^]	n.a.	n.a.	n.a.
Single-titer serology	D	Indirect evidence	n.a.	n.a.	n.a.	n.a.	n.a.	n.a.	n.a.	n.a.	n.a.	n.a.	n.a.	n.a.
Clinical diagnosis	E	Clinical diagnosis	903 (39.9)	145 (6.7)	22 (2.2)	595 (11.3)	0	0	0	0	51 (1.6)	161 (7.4)	5 (1.0)	1,882
All methods	–	–	2,263	2,162	994	5,274	297	117	921	636	3,258	2,182	509	18,613

LBRF, louse-borne relapsing fever; n.a., not applicable (PCR: method not yet available [developed 1983]; serology: no commerical test developed; thus restricted to research institutions); PCR, polymerase chain reaction.

Note: Two publications did not report cases in absolute numbers and were thus not included in the numerical analysis [44,45].

† The first publication reporting the use of PCR to characterize and identify *B*. *recurrentis* was published in 1997 [28].

‡ Between 2010 and 2019, 72 PCR-confirmed cases were reported: 2 autochthonous cases from Ethiopia and 69 imported cases from Europe (68) and Israel (1).

§ In 2000, paired serology was used to retrospectively investigate 33 LBRF cases [34] which were microscopically diagnosed in a study conducted in 1977 [46].

### Epidemiology

**Fig 4:**
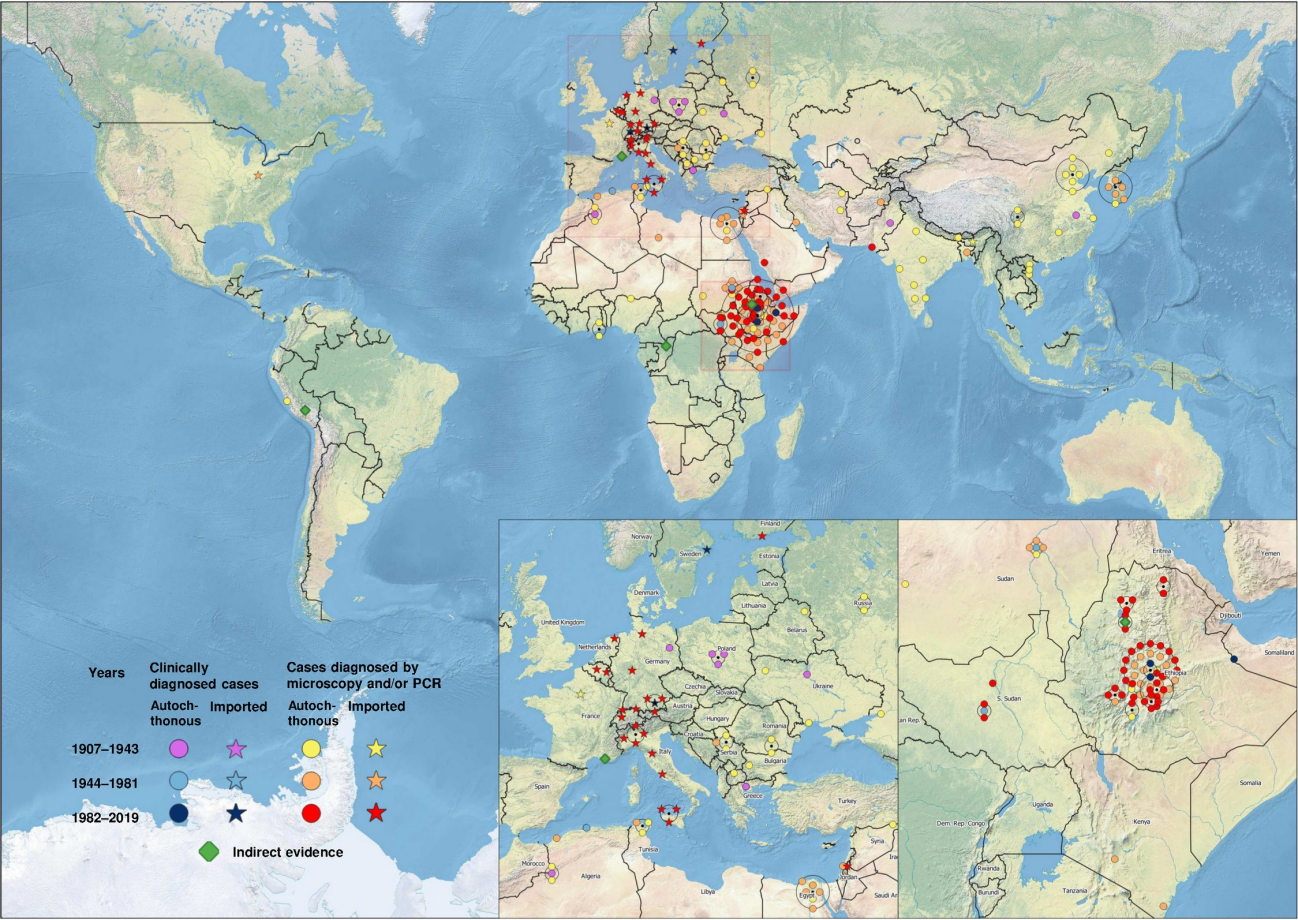
Geographic visualization of all identified LBRF cases published from 1907 to 2019. Each data point on the map corresponds to one of the analyzed 184 publications. If 2 or more studies reported cases from the same location, the dots representing these studies are connected by a circle with its center corresponding to this location. LBRF, louse-borne relapsing fever; PCR, polymerase chain reaction. (Map data: Natural Earth).

The geographic localisation of all included LBRF cases is displayed in Fig 4 according to the following criteria: (i) time of occurrence; (ii) autochthonous versus imported; and (iii) underlying diagnostic certainty.

Publications reporting only indirect evidence for the presence of LBRF (but no clinical cases) are summarized in Table 4.

**Table 4:** Publications reporting indirect evidence for the presence of LBRF.

Year	Country	Reported findings of the publication	Grd.	Ref.
1998	Peru	Detection of *B*. *recurrentis* specific antibodies in the blood of 2 out of 194 volunteers during a serosurvey conducted in rural Andean communities; based on single-titer testing; no clinical data reported	D	[30]
2000–2003	France	Detection of *B*. *recurrentis* specific antibodies in the blood of 15 out of 930 homeless people during a serosurvey conducted in Marseille; based on single-titer testing; no clinical data reported	D	[29]
2011	Ethiopia	Detection of *B*. *recurrentis* DNA in human head lice sampled; no clinical data reported	A	[47,48]
2015	Republic of Congo	Detection of *B*. *recurrentis* DNA in human head lice sampled; no clinical data reported	A	[47,48]

DNA, deoxyribonucleic acid; Grd., grade of diagnostic certainty according to Table 2; LBRF, louse-borne relapsing fever; Ref., reference.

Reports on imported cases of LBRF are summarized in Tables 5 and 6.

**Table 5:** Imported cases of LBRF reported from America, Asia, the Middle East, and Africa.

Year	Number of cases	Country of exportation	Country of importation	Comment	Grd.	Ref.
1943–1944	134	China	India	Diagnosed in Chinese soldiers after arrival in a US Army Station Hospital in Assam	B	[49]
1945–1946	64	Morocco	Senegal	In the course of 9 months, 12 ships	E	[18]
1976	1	Ethiopia	USA, Ohio	Diagnosed in an immigrant shortly after arrival; detection in blood and lice	B	[50]
1985	2	Ethiopia	Israel	Two Ethiopian immigrants	B	[51]
2015	1	Ethiopia	Israel	Priest traveling with a group of pilgrims	A	[52]

Grd., grade of diagnostic certainty according to Table 2; LBRF, louse-borne relapsing fever; Ref., reference.

**Table 6:** Imported cases of LBRF reported from Europe.

Year	Number of cases	Country of exportation	Country of importation	Comment	Grd.	Ref.
1948	1	Greece	France	Greek migrant; Migration route: unknown	B	[53]
2015	1	Eritrea	Switzerland	Eritrean refugee; Migration route: Sudan, Libya, and Italy	A	[54]
2015	15	Somalia (*n* = 12); Eritrea (*n* = 2); Ethiopia (*n* = 1)	Germany	Somali, Eritrean, and Ethiopian refugees; Migration route: Sudan, Libya, and Italy	14A, 1B	[32]
2015	2	Eritrea	the Netherlands	Eritrean refugee; Migration route: Ethiopia, Sudan, Libya, and Italy	A	[55]
2016	1	Unkown	Sweden	Migration route: unknown	E	[56]
2015	1	Somalia	Italy	Somali refugee; Migration route: unknown	A	[57]
2015	3	Somalia	Italy	Somali refugees; Migration route: Libya	A	[58]
2015	1	Somalia	Italy	Somali refugee; Migration route: Kenya, South Sudan, Sudan, and Libya	A	[59]
2015	5	Somalia	Italy	Somali refugees; Migration route: Kenya, Uganda, Sudan, and Libya	A	[60]
2015	1	Somalia	Italy	Somali refugee; Migration route: Sudan and Libya	A	[61]
2015	4	Somalia (*n* = 3), Eritrea (*n* = 1)	Switzerland	Somali and Eritrean refugees; Migration route: Sudan, Libya, and Italy	A	[16]
2016	1	Eritrea	Switzerland	Eritrean refugee; Migration route: Sudan, Libya, and Italy	B	[62]
2015–2016	25	Somalia (*n* = 23), Eritrea (*n* = 2)	Germany	Somali and Eritrean refugee; Migration route: Sudan, Libya, Yemen, and Italy	A	[13]
2016	1	Somalia	Germany	Somali refugee; Migration route: unknown	A	[63]
2015	1	Somalia	Belgium	Somali refugee; Migration route: Italy	A	[17]
2015	1	Somalia	Germany	Somali refugee; Migration route: Ethiopia, Sudan, Libya, and Italy	A	[64]
2015	2	Somalia	Belgium	Somali refugees; Migration route: Ethiopia, Sudan, and Libya	B	[65]
2014–2015	2	African region	Switzerland	Exact date and migration route: unknown	E	[66]
2017	1	East Africa	Italy	Date and migration route: unknown	A	[67]
2016	1	Mali	Italy	Malian refugee; Migration route: Algeria and Libya	A	[15]
2015	1	Somalia	Germany	Somali refugee; Migration route: Ethiopia, Sudan, and Libya	A	[68]
2015	2	Somalia	Finnland	Somali refugees; Migration routes 1: Yemen, Egypt, Greece, and Italy; 2: Uganda, Libya, Italy, and Germany	A	[14]
2016	2	Somalia	Italy	Somali refugees; Migration route: Libya	A	[69]
2016	1	Somalia	Italy	Somali refugees; Migration route: unknown	A	[70]
2014–2015	2	Somalia	Germany	Somali refugees; Date and migration route: unknown	E	[71]

Grd., grade of diagnostic certainty according to Table 2; LBRF, louse-borne relapsing fever; Ref., reference.

Reports on the occurrence of LBRF in Latin America, Asia, and the Middle East are summarized in Tables 7–13.

**Table 7:** Reports on LBRF from Latin America.

Year	Comment	Grd.	Ref.
16th century	The first appearances of LBRF may date back to the times when Spanish conquistadors arrived in South America	NA	[72]
1917	**Peru:** Retrospective description of the first microscopically diagnosed case of LBRF in Peru	NA	[72–74]
1918–1919	**Peru:** First microscopically diagnosed LBRF cases published in a study in Peru	B	[73]
around 1920	**Peru:** Relapsing fever is reported from various parts of the country, mostly from central and southern regions. The regions Ayacucho, Huancavelica, Junín, Cajamarca, Ancash, Lima, Arequipa, Cuzco, Apurímac, and Puno were reported to be affected	NA	[72,73]
1946	**Peru:** A putative case of LBRF was published in 1946, but excluded from this review because of insufficient information regarding the differentiation between TBRF and LBRF, being simply named “relapsing fever”	NA	[75]
1999	**Peru:** Detection of *B*. *recurrentis* specific antibodies in the blood of 2 out of 194 volunteers during a serosurvey conducted in rural Andean communities; based on single-titer testing; no clinical data reported	D	[30]

Grd., grade of diagnostic certainty according to Table 2; LBRF, louse-borne relapsing fever; NA, not applicable; Ref., reference; TBRF, tick-borne relapsing fever.

**Table 8:** Reports on LBRF from Northern America.

Year	Comment	Grd.	Ref.
1844–1874	Retrospective description of several epidemics of LBRF that may have occurred between 1844 and 1874	NA	[50]

Grd., grade of diagnostic certainty according to Table 2; LBRF, louse-borne relapsing fever; NA, not applicable; Ref., reference.

**Table 9:** Reports on LBRF from China.

Year	Comment	Grd.	Ref.
?	LBRF may have been around for a very long time and may have been the cause of 2 outbreaks reported from Beijing in 1864 and Hong Kong in 1865	NA	[76–80]
1904	Retrospective description of LBRF in Pakhoi in the South of China	NA	[81,82]
1905–1906	Retrospective description of LBRF in Shanghai, Tien-Tsin, Hankou, and Hong Kong	NA	[73,82]
1909	Retrospective description of LBRF in the southern region of Yunnan	NA	[72,73,82]
1911–1912	Report of an LBRF epidemic with clinically diagnosed cases in Hwaiyuan and an endemic focus in Chongqing	E; NA	[82,83]
1911–1919	Retrospective description of annual outbreaks of LBRF in Sichuan; published case series of microscopically confirmed cases from Sichuan in 1919	NA; B	[82,84]
1913	Retrospective description of LBRF among prisoners in Shanghai	NA	[82]
1913–1917	Retrospective description of LBRF in Manchuria until 1917; published notes on microscopically confirmed cases from an outbreak in a mine in 1913	B	[82,85]
1918–1938	Report of sporadic cases and occasional small outbreaks of LBRF in Hunan, including 41 microscopically diagnosed cases	B	[79]
1919	Retrospective description of LBRF cases among soldiers in Fujian	NA	[77,82]
1920	Descriptive note that relapsing fever had been found at any location in China where laboratories had been built	NA	[82]
1931	Reports of microscopically diagnosed LBRF cases in Beijing	B	[77]
1932	Retrospective description of endemic LBRF in all provinces along the Yangtse River and sporadic epidemics. Further notes dating back to 1924 and 1925 indicate the presence of LBRF in Tibetan regions from where the disease spread both along the eastern trading route toward Tachienlu (today Kangding) and the southern trading route along the Mekong river	NA	[76]
1932	Report on a minor epidemic with microscopically diagnosed cases of LBRF in Shanghai (including the note of the disease’s constant presence in the hospital records over the past 25 years)	B	[76]
1936–1939	Several reports on microscopically diagnosed LBRF cases in Beijing	B	[80,86–89]
1943–1944	Report of microscopically diagnosed LBRF cases in Chinese soldiers just flown into Assam, India. The infections were considered to have been acquired in China	B	[49]

Grd., grade of diagnostic certainty according to Table 2; LBRF, louse-borne relapsing fever; NA, not applicable; Ref., reference.

**Table 10:** Reports on LBRF from Korea and Japan.

Year	Comment	Grd.	Ref.
?–1913	Notes of publications in Japanese about LBRF cases in Japan. Description of the presence of LBRF in Tokyo during 1913	NA	[85]
?–1913	LBRF is believed to have been clinically diagnosed before 1913 in Korea	NA	[90]
1913–1943	Report of the first microscopical diagnosis in 1913; notes of repeated incidence in local hospital admission records; description of an epidemic among railway laborers and of accounts in other languages such as Korean or Japanese	B; NA	[90]
1950–1955	Several reports on microscopically diagnosed LBRF cases during the Korean War, especially among Chinese and Korean prisoners of war or United Nations personnel. The presence of LBRF in the native population is noted. Movement of Chinese troops is suggested to have imported and perpetuated the disease in some regions	B	[91–95]

Grd., grade of diagnostic certainty according to Table 2; LBRF, louse-borne relapsing fever; NA, not applicable; Ref., reference.

**Table 11:** Reports on LBRF from Southeast Asia.

Year	Comment	Grd.	Ref.
?–1906	LBRF may have been around for a long time, but has been confused with malaria in Vietnam	NA	[96]
1907–1912	Report of microscopically diagnosed LBRF cases in Hanoi. The disease has been reported yearly in hospital statistics since 1907; in 1912, case numbers began to rise	B	[97]
1908	Restrospective description of 4 Chinese LBRF patients from Yunnan treated in Hanoi	NA	[82]
1907–1909	Retrospective description of cases in the province Thanh-Hoa	NA	[96]
1912	Reports of microscopically diagnosed LBRF cases in the provinces of Thanh-Hoa and Nghê-An in 1912. Retrospective descriptions of clinically suspected cases in earlier years, endemic foci in these and more southern reagions, such as Ha-Tinh	B	[96,98]
1950–1958	Retrospective descriptions of cases in Cambodia between 1950 and 1958	NA	[25]

Grd., grade of diagnostic certainty according to Table 2; LBRF, louse-borne relapsing fever; NA, not applicable; Ref., reference.

**Table 12:** Reports on LBRF from the Indian subcontinent.

Year	Comment	Grd.	Ref.
18th century	LBRF may have existed on the Indian subcontinent since the mid-18th century	NA	[81]
1836–1877	Retrospective description of severe epidemics in the United Provinces in 1836, 1837, and 1862; retrospective description of the first recognized outbreak in 1852 in Usufzai Valley, in present day Pakistan; retrospective description of an epidemic in Patna in 1856 and Bombay in 1877; retrospective description of an epidemic in the Réunion due to infected coolies shipped from said epidemic in Bombay	NA	[81,99,100]
1869–1911	Retrospective description of endemic foci in northern India and the Himalaya region with several minor outbreaks and occasional epidemics, such as in Punjab and the United Provinces (in 1869, 1878, 1891, 1896, 1899, 1906, and 1911)	NA	[81,99]
1905	Report of microscopically diagnosed LBRF cases in an epidemic in Peshawar Valley, in present day Pakistan	B	[101]
1906	Report of an epidemic in Sirur with notes of microscopically diagnosed cases	B	[102]
1907	Report of microscopically diagnosed LBRF cases and first description of the mode of transmission and discovery of *B*. *recurrentis* in lice	B	[7]
1908–1911	Report of microscopically diagnosed LBRF cases in Bulandshahr. It further suggests the yearly occurrence, but also a certain neglectance of the disease, being often misdiagnosed for malaria in the area	B	[103]
1911	Report of microscopically diagnosed cases in Bangalore	B	[39]
1912	Report of microscopically diagnosed cases in Darjeeling District	B	[104]
1917–1920	Description of an LBRF epidemic affecting Punjab, the United Provinces, the Central Provinces; report of microscopically diagnosed cases during this epidemic in the Seoni District (Central Provinces). Description of the presence of LBRF before the epidemic in these areas	NA; B	[99,105]
1923	Report of microscopically diagnosed LBRF cases in Raichur	B	[106]
1923–1924	Retrospective description of an epidemic in Madras	NA	[107]
1923–1926	Account on diseases in India suggesting LBRF to be far more widely spread at the time than formerly suspected, with affected areas scattered in both central and southern areas with a few spots in eastern areas	NA	[108]
1924	Description of microscopically diagnosed cases in Nilgiri Hills, Madras Presidency, and further research with sera of patients	B	[107,109]
1925–1929	Description of sporadic cases and the occurrence of small outbreaks in Punjab between 1925 and 1929, indicating endemic residua in the North after the epidemic. Following the description of sporadic LBRF cases in the North-West Frontier, in present day Pakistan, the report further suggests both the louse-borne and the tick-borne variety to be endemic in these areas	E	[107]
1948	Report of microscopically diagnosed LBRF cases in North-East Bengal among military personnel	B	[110]
1984–1985	Report of 2 microscopically diagnosed LBRF cases in the Karachi region. Finding was called “*B*. *recurrentis”* by authors; however, the publication lacks data on differentiation	B	[111]

Grd., grade of diagnostic certainty according to Table 2; LBRF, louse-borne relapsing fever; NA, not applicable; Ref., reference.

**Table 13:** Reports on LBRF from the Middle East and Afghanistan.

Year	Comment	Grd.	Ref.
?–1950	Report of microscopically diagnosed LBRF cases in Kabul. The disease was said to be frequently encountered in Afghanistan	B	[112]
?–First World War	LBRF may have been around for a long time. Descriptions of LBRF being endemic among the native population	NA	[113–115]
First World War	Description that Baghdad and the northern Persian areas were most affected, as well as Birjand, Busra, Meshed, and several other locations	NA	[113,114,116]
1918	Report of microscopically diagnosed LBRF cases in Meshed, Birjand, and a railway construction camp near Nisibin (today Iran). Many of the occurrences were traced back to troop movements, close contact with the native population, or recruiting of refugees for labor corps	B	[114,117,118]
1945–1946	Report of microscopically diagnosed LBRF cases during an epidemic in the aftermath of the Second World War in Abadan. Authors discussed former putative LBRF epidemic during the Second Word War mislabelled as “typhus” and further highlight the presence of TBRF in the area	B	[119]

Grd., grade of diagnostic certainty according to Table 2; LBRF, louse-borne relapsing fever; NA, not applicable; Ref., reference; TBRF, tick-borne relapsing fever.

**Fig 5:**
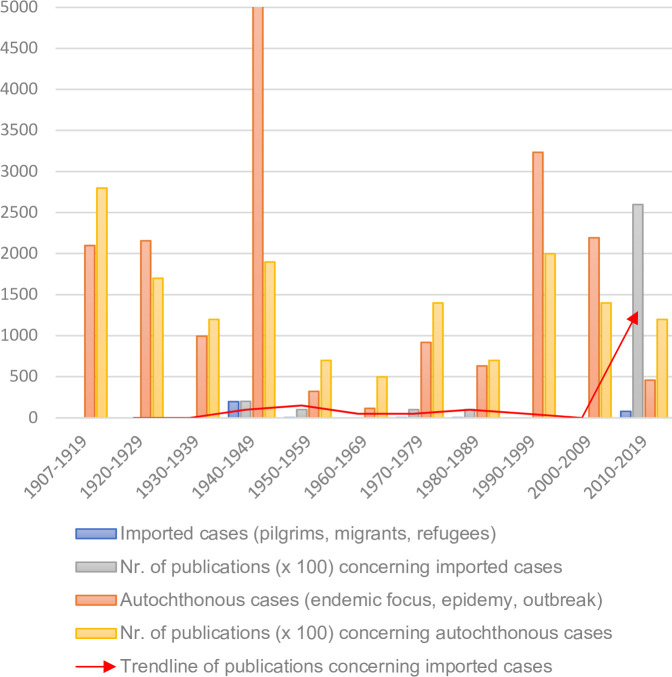
Number of published LBRF cases in relation to the number of publications. For better visualization, the number of publications is multiplied by 100. The red arrow depicts the trendline of publications reporting imported cases of LBRF. LBRF, louse-borne relapsing fever.

In Fig 5, the number of published LBRF cases is visualized in relation to the number of publications.

### Diagnostic aspects

Tables 14–17 summarize the milestones of LBRF diagnostics.

**Table 14:** Milestones in microscopy.

Year	Comment	Ref.
1873	Discovery of the causative organism	[6,120]
1907	Discovery of the organism in the vector	[7]
1947	A publication reported 65% of suspected relapsing fever cases to yield a positive result on microscopic examination	[121]
1969	A retrospective review of 2,825 cases of relapsing fever suggested a sensitivity of approximately 70% on initial blood smears to find spirochetes	[36]
1983	Enhancement of sensitivity was reported when using either fluorescence microscopy on acridine orange-stained blood smears	[122]
1996	A review suggested animal inoculation or culture to enhance sensitivity and specificity	[27]
2001	Enhancement of sensitivity was reported using QBC fluorescent technique. (QBC: detection of spirochetes down to 10 organisms/mm3, Wright-stained blood film: did not detect organisms <82 organisms/mm3; positive readings found by means of blood film fell significantly after dilutions below 3,263 organisms/mm3, in contrast to the QBC, the accuracy of which fell only at dilutions of <82 organisms/mm3)	[123]
2009	A publication suggested a centrifugation-based method to concentrate spirochetes, followed by Giemsa staining, to be equally sensitive to PCR-based methods. According to the study results, detection at a bacterial concentration of 1 bacteria/ml was achieved	[124]

PCR, polymerase chain reaction; QBC, quantitative buffy coat; Ref., reference.

**Table 15:** Milestones in serology.

Year	Comment	Ref.
?–2019	Antigenic variation and cross-reactivity within the species, and with other closely related organisms, were 2 major issues in the development of reliable serological diagnostic methods	[25,34,38,125–128]
1996 and 2000	Publications suggest the use of *glpQ* gene, which was found to be absent in Lyme disease borreliae, and very different in amino acid sequences in *Treponema pallidum*, hence enabling the serological distinction from relapsing fever cases	[34,127]
2000	A report demonstrated seroconversion 1 to 2 weeks after clinical presentation, hence recommending the use of paired acute-phase and convalescent-phase sera. Persistence of detectable antibodies to recombinant GlpQ in a serum sample taken 27 years after infection with LBRF. Immunoblotting with recombinant GlpQ was found to be more sensitive than ELISA with purified His-tagged GlpQ	[34]

ELISA, enzyme-linked immunosorbent assay; GlpQ (protein)/glpQ (gene), glycerophosphodiester phosphodiesterase; LBRF, louse-borne relapsing fever; Ref., reference.

**Table 16:** Milestones in PCR.

Year	Comment	Ref.
1996	Phylogenetic analysis, using PCR-based methods targeting 16S ribosomal DNA sequences, reported a close relationship between the different relapsing fever borreliae, and researchers have suggested this strategy for diagnosis	[129]
2003	Report of successful discrimination using a real-time PCR assay targeting the flagellin gene in *B*. *recurrentis*, which differs only by a single nucleotide from the sequence in *B. duttonii*. Successful recognition has been achieved at annealing/extension temperatures of 64.5°C, 65°C, and 66°C. Sensitivity of 3 copies of the target sequence was reported	[41]
2008–2012	Phylogenetic analysis has led to the concept that *B*. *recurrentis* is a degraded subset of *B*. *duttonii*. Using a MLSA approach, gene sequence identities greater than 99% were reported	[130–132]
2012	Using MST method, 3% sequence divergence was observed when using the MST7 spacer to discriminate between *B*. *duttonii* and *B*. *recurrentis*	[131]
2013	Development of an MR-TPCR assay reporting a 100% sensitivity and specificity for both *B*. *duttonii/recurrentis* and *Borrelia hispanica*, as well as a 99% sensitivity and specificity for *Borrelia crocidurae*. 16S rRNA gene probe for the detection of any relapsing fever borreliae, combined with species-specific primers (recN gene detecting *B*. *duttonii/B*. *recurrentis*). Accuracy of detecting 100 copies was reported. Successful discrimination between *B*. *duttonii* and *B*. *recurrentis* was not achieved	[42]

DNA, deoxyribonucleic acid; MR-TPCR, multiplex real-time PCR; MSLA, multilocus sequence analysis; MST, multispacer sequence typing; PCR, polymerase chain reaction; Ref., reference; rRNA, ribosomal ribonucleic acid.

**Table 17:** Milestones in animal inoculation and culture.

Year	Comment	Ref.
1954	The inoculation of guinea pigs was suggested for use in differential diagnosis of TBRF and LBRF: Adult rodents are susceptible to TBRF borreliae, but refractory to *B*. *recurrentis* infection	[120]
1958 and 1969	Susceptibility tests in adult rodents were reported to be the most reliable method for the differentiation between tick-borne and louse-borne borreliae	[133,134]
1965 and 1968	Reviews on LBRF describing that monkeys are susceptible to infection with *B*. *recurrentis*, while adult mice and rats have limited susceptibility. Young mice and rats were found to be susceptible	[125,135]
1969 and 1971	Reviews retrospectively describing that maintenance and arguably even limited growth of *B*. *recurrentis* was achieved in vitro	[25,36]
1971	Monograph describing animal inoculation with *B*. *recurrentis* on various animals with differing results regarding their susceptibility until 1971, pointing out lack of details in many reports which limits the comparability	[25]
1984	Retrospective description of the successful multiplication of *Borrelia hermsii* in 1971 on a media, which led to the creation of the BSK II medium in 1984 (originally used to cultivate *Borrelia burgdorferi*)	[136]
1994	Report of the first growing isolate of *B*. *recurrentis* in BSK II medium	[137]
2009	Report showing improved results using immunodeficient mouse strains	[138]

BSK, Barbour Stoenner Kelly; LBRF, louse-borne relapsing fever; Ref., reference; TBRF, tick-borne relapsing fever.

## Discussion

### Epidemiology

#### Latin America

The last conclusive evidence of the occurrence of LBRF in Latin America dates back almost a century and is restricted to Peru. This notably contrasts the assumed occurence of the disease in South America in the mid-20^th^ century published by Felsenfeld in 1971 (Fig 1) (Note: The method used to create the map is not known to us; the question marks within the map suggest some degree of uncertainty). We did not find any evidence supporting the persistent occurence of LBRF foci in Peru beyond the 1920s, nor did we find any reports on the occurrence of LBRF in other countries of Latin America.

#### Asia and the Middle East

Reports from Asia often coincide with times of colonialization as well as wars that western countries were involved in, when medical officers published their observations during their service. Subsequently, publications ceased shortly after the ends of conflicts and colonialization. The most recent examples were the Second World War and the Korean War. However, endemic foci may have persisted among a certain population at risk, as it currently does in Ethiopia, which simply were not detected or not published in western languages. It seems unlikely that the disease vanished as abruptly as publications ceased. A publication bias seems likely for these regions. However, our findings strikingly contrast the assumed occurence of LBRF in Asia in the mid-20th century published by Felsenfeld in 1971 (Fig 1).

China: The last published report dealing with microscopically diagnosed LBRF cases linked to China dates back to 1946, when relapsing fever was reported in Chinese soldiers just flown into Assam, India (Table 9). No reports on cases of LBRF exist from China thereafter.

Korea and Japan: Scarce data are available from Korea, where LBRF occurred and was confirmed until the end of the Korean War (Table 10). A retrospective description suggests a far wider spread than assumed in English literature and describes existing publications in Japanese or Korean language before the Korean War [90]. Thereafter, no information is available.

Southeast Asia: Data from Southeast Asia, where LBRF was reported in Vietnam and Cambodia, are very limited, and no information is available after 1912 and 1958, respectively (Table 11). Remaining residua past that can neither be confirmed nor excluded.

Indian subcontinent: Many accounts were published from the Indian subcontinent, where the disease often occurred in epidemics and endemic foci (Table 12). Investigations in the North-West Frontier province, nowadays located in Pakistan, led to the belief that both the louse-borne and the tick-borne variety are endemic in these areas, suggesting that careful watch should be kept for a sufficient differentiation of LBRF to TBRF [107]. The diagnosis of the reported sporadic cases was established after careful consideration of TBRF as a differential diagnosis. The author notes further that in most cases, blood was taken after symptoms resolved. Therefore, most microscopic examinations were negative. As there was no further data on the positive results, all cases were considered as clinically diagnosed. A worker informed the authors in a personal communication that LBRF had been known in the area for many years [107]. The last published report from 1990 microscopically diagnosed LBRF in 2 patients in 1984 to 1985 [111]. Despite the authors titling this discovery *B*. *recurrentis*, there is neither sufficient clinical evidence nor conclusive case histories to retrospectively comprehend how the differentiation was achieved. The authors noted that the disease is common in the Karachi region; however, their work lacks discussion as to whether these cases belong to the tick-borne or louse-borne species [111]. The study was included due to positive blood smears and the authors publishing the data as *B*. *recurrentis*. However, considering the circumstances, the evidence should be regarded with caution. Further studies are needed from this area to either confirm or rebut the presence of LBRF in these areas.

Afghanistan and Middle East: Accounts on LBRF from former Persia and Mesopotamia are closely related to the World Wars and the medical officers who published their reports, with no further information published thereafter (Table 13). Migration of refugees, prisoners of war, and the movement of Russian, Turkish, and Indian troops were often reported to be the main cause for the infection of western troops with the disease [113,114,116]. Many of the occurrences mentioned were traced back to troop movements, close contact with the native population or the recruiting of refugees for labor corps [113,114,116–118]. A report from Iran in 1976 found borreliae in a febrile patient, suggesting a new species in the area, although noting a similarity to the malady known from Ethiopia. Despite the suspicions, no further research on the means of transmission has been conducted. For this reason, the species of borreliae found in Iran remains inconclusive [139]. Caution should be taken regarding sporadic cases, as the authors themselves and others noted the presence of TBRF in these areas [119,139].

#### Imported cases

Living in cramped and poor hygienic conditions provides favorable conditions for the transmission of LBRF [19,20,45,134,140–145]. As this is the case for many immigrants, refugees, and seasonal workers, human migration is a critical component in the development of LBRF epidemics. In the last century, when the presence of LBRF was almost worldwide, only sporadic cases of imported LBRF were reported. In recent years, the proportional discrepancy between the number of reports from endemic regions and reports on imported cases to non-endemic countries is striking: Between 2010 and 2019, 25 publications reported 78 imported cases of LBRF in non-endemic countries, whereas from endemic regions, 7 publications reported 431 autochthonous cases (Fig 5). This increase in reports on imported cases of LBRF is primarily attributable to the increased migration flow from Africa to Europe observed in 2015 and 2016. Most of these cases were diagnosed using PCR-based methods.

#### Persistence of LBRF and factors perpetuating the disease

The vector: The transmitting vector *P*. *humanus* [146] is a specialized human ectoparasite that flourishes in hygienically poor and overcrowded conditions [11,146]. Besides *B*. *recurrentis*, it also transmits *R*. *prowazekii* and *B*. *quintana*, the causative agents of epidemic typhus and trench fever, respectively [146]. Experiments demonstrated that *B*. *recurrentis* is not transmitted by ticks [147]. The body louse is currently the only proven vector, and humans are the only known reservoir [11]. Body lice live and lay eggs in clothing and only approach the human body for an obligate blood meal [40]. A factor that perpetuates transmission, especially during epidemics, is that lice are temperature sensitive and tend to leave patients clothes during febrile episodes [22]. Interestingly, body lice were reported on secondhand clothing found on a street market in Italy in 2018, making it the first report of human body lice since 1945 in Italy [148]. This finding challenges the paradigm that body lice die quickly once off the host.

Transmission: Unlike most vector borne infections, *B*. *recurrentis* is not transmitted to the human host during the blood sucking act of the vector, as the digestive tract and the salivary glands of the body louse are not affected by the infection [11,86,149,150]. Since the louse’s hemolymph was found to harbor *B*. *recurrentis*, human infection is traditionally considered to result from damaging or crushing the louse, thus liberating the insect’s hemolymph. The co-liberated bacteria were then considered to enter the human host through microlesions of the skin, which are either caused by the bite itself or by scratching induced by the itchiness of the bites [11]. This transcutaneous route of infection is supported by animal [80] as well as human experiments [86,151]. In 1938, Chung and colleagues identified *B*. *recurrentis* in the feces of lice and suggested this as an additional source of infection, with the caveat that in their experiments, the excreted spirochetes were dead [86]. The issue remained dormant until 2005, when Houhamdi and Raoult reported the detection of living *B*. *recurrentis* in excreted feces of an infected louse, which revived the discussion [152]. However, whether the fecal excretion of *B*. *recurrentis* is relevant for the pathogens transmission still remains to be clarified.

#### Unnoticed reservoir?

Asymptomatic cases: Asymptomatic cases of LBRF in Sudan were observed by Atkey in 1930 [153]. These cases appeared toward the end of an epidemic, and spirochetes were readily found in their blood. The author further observed a general milder course of the disease toward the end of the epidemic [153]. Another author reported latent and atypical LBRF infections [154]. During an outbreak survey conducted in Khartoum in 1969, 22 microscopically positive but asymptomatic cases of LBRF were identified among 979 immigrant laborers from southern Sudan [134]. The fact that such cases have been reported may imply a high number of overlooked cases. Further research is needed to confirm the existence of asymptomatic cases and to assess the rate of asymptomatic infections since they may contribute to the persistence of the disease in certain areas.

Residual brain infection: Residual brain infection (RBI) describes the tendency of spirochetes to persist in the brain after they have cleared from the blood and was first described by Buschke and Kroo with TBRF in an animal model [38]. Data for *B*. *recurrentis* are scarce. One study demonstrated infection of squirrels using cerebrospinal fluids of LBRF patients [87]. Other studies demonstrated *B*. *recurrentis* in the cerebrospinal fluid of patients [155] and infection of the brain of primates [121]. The involvement of the central nervous system in the acute phase of LBRF infection was suggested in up to 30% of cases [36]. However, a review article suggested that most of neurologic symptoms in LBRF infection may be due to hemorrhages in the central nervous system, rather than direct involvement of the spirochetes [38]. In animal experiments with *Borrelia turicatae*, RBI was found in 19% of immunocompentent mice [156]. Another study using a murine model to investigate RBI using *B*. *turicatae*, *Borrelia crocidurae*, *Borrelia hermsii*, and *B. duttonii* found limited brain persistence in *B*. *crocidurae* and longest persistence for *B*. *duttonii*. Additionally, reactivation of the infection was demonstrated in the case of immunosuppression. The authors suggested the brain as reservoir [157]. Further studies are needed to investigate RBI.

Historic point of view: Many hypotheses were discussed regarding the question of persistence and maintenance of the disease between large epidemics. As an example, in North Africa, despite systematic research, no LBRF cases were identified between the 2 large epidemics of 1908 to 1920 and 1943 to 1945 [2]. Considering that LBRF has no currently known host other than humans, that there is currently no known reservoir, that lice do not transmit *B*. *recurrentis* to their progeny, and that transmission requires injury or crushing of the louse, the persistence of the disease was a mystery. Two hypotheses were formulated around 1960 concerning the origins of epidemics: (i) the epidemics of LBRF are only a temporary phenomenon due to a mutation of a tick-borne species by passage through the human into the louse; and (ii) the existence of reservoirs in an endemic focus from where epidemics originate [2]. Regarding the first hypothesis, it was experimentally achieved to infect lice with tick-borne species [2]. In one study, lice were infected with *B*. *duttonii* and successfully transmitted back to primates [158]. However, no mutations or changes in pathogenic character were observed, and no evidence supporting this hypothesis was found in vivo. Regarding the second hypothesis, Ethiopia was recognized to be an endemic focus by Sparrow [133]. Since then, Ethiopia has remained an endemic focus. It seems likely that the persistence of endemic foci were the origins of the former epidemics, rather than the first mentioned, or any other hypothesis. Asymptomatic infections and RBIs most probably serve as factors that further perpetuate the persistence of LBRF in an endemic focus.

#### New endemic foci?

Most of the recently imported cases originated from the Horn of Africa. In one refugee from Mali, contact with people migrating from this area was reported. The location of a current endemic focus has been suspected in Libya, which reportedly serves as a focal point for smugglers to bring refugees across the Mediterranean Sea [15,32,159]. Even though there is no published evidence to confirm the suspicions, a temporary unnoticed endemic focus in certain refugee camps or places where migrants have gathered is likely. The duration of a migrant’s journey from East Africa to Europe largely exceeds the reported incubation periods of LBRF. It is probable that refugees go through the first attack and the following relapses during the first month of their journey. The fact that these patients have showed first symptoms upon arrival in Europe suggests an endemic focus around the Mediterranean Sea. This could have been a temporary focus, as it vanished after 2016 without further reports from imported cases thereafter. However, the epidemiological investigation in this review has shown that there is currently no other known endemic focus than Ethiopia.

#### Migrants and the homeless population, overlooked small outbreaks in Europe?

Since the Second World War, there has been no reported outbreak of LBRF in Europe. In 2005, one study indicated that there may have been a small outbreak among the homeless population in Marseille based on the detection of immunoglobulin G (IgG) antibodies to *B*. *recurrentis* [29]. In Marseille, about 60% of the homeless population notably consists of migrants [29,160]. It is possible that an imported, unnoticed case could have caused a small epidemic among the high-risk population of homeless people. According to several studies, these people are commonly found infested with body lice, and in some instances further infected with other louse-borne pathogens, such as *B*. *quintana* [161–164]. A case report from Saudi-Arabia described *B*. *recurrentis* in a homeless man [165]. A report from Italy suggested that 2 migrants acquired the disease in a housing facility of newly arrived refugees. The authors noted that the disease was only diagnosed because of the microscopic blood smear investigations for malaria conducted due to the patients’ recent migration history. They further stated that febrile patients without travel history may receive empirical antibiotic treatment, which may result in the resolution of symptoms without further investigation [60]. Given the susceptibility of borreliae to common empirical antibiotic treatment and the unspecific symptoms of the disease, such minor outbreaks may be easily overlooked. Moreover, vulnerable population groups, such as the homeless, may have limited access to medical care. Under these premises, the introduction of a single case into such surroundings may be sufficient to create a small, unnoticed outbreak.

Thus, in regard to LBRF, close attention should be paid to patients from vulnerable population groups, such as migrants or the homeless that display febrile symptoms. Considering asymptomatic cases, publication bias, and possible temporary foci somewhere along migration routes, another reemergence of the disease should neither be neglected nor its epidemic potential be underestimated.

#### New vector?

Interestingly, two studies found DNA of *B*. *recurrentis* in head lice obtained from humans in the Republic of Congo and Ethiopia [47,48]. They raise the question whether head lice can transmit human louse-borne pathogens. The evidence suggests that head lice, contrary to former belief, may act as a vector. However, further research is necessary to investigate the role of head lice in the transmission of louse-borne pathogens.

### Diagnostic aspects

#### Microscopy

Microscopy remains the gold standard for diagnosing LBRF since the discovery of the organism in 1867. The sensitivity, however, is directly affected both by the number of spirochetes in the blood and whether the blood sample was taken during a febrile or afebrile period of the infection. Blood should be obtained during a febrile period [19,36–38,124], yet it is possible, though very hard, to find spirochetes during afebrile periods [124]. Although data on sensitivity are scarce, one study reported spirochetes in 38% of patients whose blood had been taken during an afebrile period [155], and another study reported positive microscopy in 5% during afebrile periods [27]. The number of positive results may be increased through the examination of repeated blood smears [36]. Furthermore, the results are often dependant on the fixative and staining method used [25]. Additionally, the level of experience the observer has is another issue that can influence sensitivity [166]. Five cases were identified that reported negative microscopy but positive PCR. In one case, blood was taken the day after initiation of adequate empirical antibiotic therapy [57], and in the other four, reasons remain unknown [69,167]. One study reported positive blood smears, but only after reexamination by an experienced microbiologist [65]. The sensitivity of 70% reported in 1969 was obtained through analysis of both tick-borne and LBRF cases [36], hence may not be fully representative for *B*. *recurrentis* specifically. Research into the validation of the enhancement methods is needed and has already been suggested [136].

#### Animal inoculation, serology, and PCR

Animal vaccination has been used to aid diagnosis, for research purposes and to some extent as a tool to differentiate between TBRF and LBRF. However, animal inoculation has never been routinely used for diagnostic reasons alone. Lack of standardized data and protocol is further limiting this method [25]. Historically, serological methods were extensively investigated, but the development was hampered by antigenetic variability and cross-reactivity [25,27,34,38,125–128]. To date, serology is not routinely used for diagnosis and is not recommended. PCR-based methods lack availability in poor countries, however, are unquestionably the best and arguably only means for a certain diagnosis of LBRF. Still, protocols for differentiation of *B*. *recurrentis* and *B*. *duttonii* need to be established. Due to the proximity of these relapsing fever borreliae, development of specific diagnostic tools and accurate discrimination between the species are challenging [131].

Considering that roughly 90% of all published LBRF cases have been diagnosed by microscopy and that microscopy continues to be the most widely used and available diagnostic method, there is a need to determine its sensitivity and to evaluate how to increase sensitivity by serial investigations and/or enhancement methods. Negative microscopy results should be regarded critically, since the last available sensitivity data from 1969 suggested a sensitivity of 70% on a single blood smear. Positive microscopy should be regarded critically, especially in areas where TBRF is endemic. The data show that microbiological methods were mainly used in Europe. In Ethiopia, the country most affected by the disease, microscopy remains the main diagnostic tool.

**Key Learning Points**

- Currently, East Africa remains the only endemic focus of louse-borne relapsing fever (LBRF).
- Human migration has repeatedly imported the disease into non-endemic countries.
- Although polymerase chain reaction (PCR)-based methods are the only means of species identification, microscopy remains the gold standard in diagnosing LBRF.

**Top Five Papers**

1. Warrell DA. Louse-borne relapsing fever (Borrelia recurrentis infection). Epidemiol Infect. 2019;147:e106. doi: 10.1017/S0950268819000116
2. Bryceson ADM, Parry EHO, Perine PL, Warrell DA, Vukotich D, Leithead CS. A clinical and laboratory study of 62 cases in ethiopia and a reconsideration of the literature. QJM. 1970;39(1):129–70. doi: 10.1093/oxfordjournals.qjmed.a067198
3. Felsenfeld O. Borrelia: Strains, Vectors, Human and Animal Borreliosis. St. Louis: Warren H. Green; 1971.
4. Southern PM, Sanford JP. RELAPSING FEVER: A Clinical and Microbiological Review. Medicine. 1969;48(2):129–50. 00005792-196903000-00002.
5. Goubau PF. Relapsing fevers. A review. Ann Soc Belg Med Trop. 1984;64(4):335–64. WOS:A1984ABX2000002.

## Supporting information

S1 Checklist PRISMA Checklist. Twenty-seven-item checklist for systematic reviews. PRISMA, Preferred Reporting Items for Systematic Reviews and Meta-Analyses.
(DOC)

S1 Text Review protocol. Established to conduct this systematic review.
(DOCX)

S2 Text Data extraction sheet. Used for screening and selecting eligible publications.
(DOCX)

S3 Text References. Reference list of included and excluded publications.
(DOCX)

S1 Fig PRISMA flow diagram. (PDF)

S1 Data Data extracted from included studies. Excel spreadsheet containing, in separate sheets, the underlying numerical data.
(XLSX)
