# Supplementary material for: Louse-borne relapsing fever—A systematic review and analysis of the literature: Part 1—Epidemiology and diagnostic aspects
Source: PLoS Negl Trop Dis. 2021 Mar 11;15(3):e0008564. doi: 10.1371/journal.pntd.0008564 (PMC7951878; doi:10.1371/journal.pntd.0008564)
Supplement: S1 Text — Established to conduct this systematic review. (DOCX) [file pntd.0008564.s002.docx]

S1 Text. Systematic Review Protocol

Inhaltsverzeichnis

[Review Title: 1](#_Toc493497631)

[Reviewer roles 1](#_Toc493497632)

[Centre conducting the review: 1](#_Toc493497633)

[Background: 2](#_Toc493497634)

[Results of Scoping searches/summary of existing literature: 3](#_Toc493497635)

[Study rationale: 4](#_Toc493497636)

[Review questions/objective: 4](#_Toc493497637)

[Inclusion criteria: 5](#_Toc493497638)

[Outcomes, intervention and phenomena of interest: 6](#_Toc493497639)

[Types of studies: 6](#_Toc493497640)

[Search and selection strategy: 6](#_Toc493497641)

[Assessment of methodological quality: 7](#_Toc493497642)

[Data synthesis, discussion and context: 8](#_Toc493497643)

[Timetable: 8](#_Toc493497644)

[Authors: 9](#_Toc493497645)

[Table 1: Screening and Selection Tool 4](#_Toc493496727)

# Review Title:

What is the current state of knowledge concerning epidemiology and the geographical spread of LBRF? What are Risk Factors to acquiring LBRF, what are Risk Factor for fatal outcome and what is the economic impact of LBRF? What is the clinical impact of LBRF?

# Reviewer roles

Primary reviewers: Pascal Kahlig and Daniel H. Paris
Secondary reviewers: Andreas Neumayr
Quality assessors: Daniel H. Paris and Andreas Neumayr

# Centre conducting the review:

Swiss Tropical and Public Health Institute, Department of Medicine
University of Basel, Faculty of Medicine

# Background:

Louse borne relapsing fever is a disease caused by B. recurrentis, transmitted by the human body louse (Pediculus humanus humanus). Between 1485 and 1551 five epidemics known as the “English sweat” occurred in London believed to be caused by “typhus”, at those times the term did not discriminate between the causing agents nowadays known, such as Rickettsia or Borrelia (D.Paris-Buch Doktorarbeit-Quellle finden sobald Herr Paris zurück ist) First use of the term “Relapsing fever” was following an outbreak of infection in Edinburgh during 1843-1848. In history, the disease had a massive impact, especially following political crisis and warfare. In the USA reports of relapsing fever date back to 1844, with an outbreak reported among immigrants from Liverpool in Philadelphia. An estimated 1’570’604 cases were believed to have occurred in the former “European Russia” during 1920 alone. (1) During World War II 400’000 cases were observed in Algeria, 400’000 in Tunisia, 180’000 in Morocco and 1’300’000 in Egypt. (2) With the general worldwide demise of the Pediculus humanus humanus, declines of LBRF have been observed. Nowadays most infections are being reported from Ethiopia and its surrounding countries. (1) In this region, it remains the seventh most common cause for hospital admission (2.5% of total; 3’777 cases) and the fifth most frequent cause of death (0.9%, 42 cases), being largely perpetuated by poverty (1) (2) (3) Furthermore there are reports of louse infestation among certain groups as the homeless, refugees and even travellers which coupled with the increased human globalization may lead to an expansion of infections from its current focus. (2) (4)
Additionally, the current political and migrational situation in eastern Africa and at the Horn of Africa, results in big numbers of people seeking protection in those countries and beyond. (5) These situations inevitably lead to camps with poor hygiene and overcrowding.

Until recently, the transmission has been considered only by damaging or destroying the louse and hereby releasing the infectious haemolymph of the haemocoel. This resulted in the assumption that during a normal feast of the body louse, B. recurrentis is not transmitted, as the digestive tract and the salivary glands aren’t affected by it. However, this dogma now has been challenged by Houhamdi and Raoult, who demonstrated that B. recurrentis is excreted in faeces of an infected louse. The same route that has been described for the Rickettsia Prowazekii and Bartonella Quintana. (6)
For now, humans are the only known reservoir of B. recurrentis and the human body louse being the only proven vector. Currently there is no evidence that infected lice transmit to their progeny, unlike the ticks. (2)

The illness can be severe with a mortality rate of up to 70% in outbreaks. (7) (8) Other reports mention a significantly lower mortality rate up to 30% of untreated cases. However, this can be reduced to 2% - 6% with appropriate treatment. (2) It appears that several factors collectively assist to the prolonged survival within blood, causing the recurrent febrile episodes, such as the gene conversion, leading to multiphasic antigenetic variation or the ability to bind factor H and factor-H-like proteins. (2) LBRF is endemic in several highland regions in Africa, South America and Asia, especially in overcrowded regions and under poor hygienic conditions. (9) (8) However, a recent publication by SJ Cutler et al. suggests that despite its epidemic potential, the disease currently persists only in Ethiopia, occasionally spilling into neighbouring countries, possibly in association with the recurrent famines and drought affecting these areas. A definite current spread has not yet been clarified. The adaptation of B. recurrentis may represent an evolutionary bottleneck, resulting in the recently observed demise of LBRF. (2)

Diagnosis typically relies on Microscopy, searching for the Spirochaetae in dark field or phase contrast microscopy. (8) Molecular Diagnostics or MOLDI – TOF are recommended to clearly identify the Bacteria, as the Microscopy is unable to discriminate between the different types of Borrelia that cause Relapsing Fever. Often only Microscopy is used and assumption to the causative species is based on geographical location and vectors. (2) (3) Serology and blood culture are not recommended due to currently being unreliable. It is assumed that B. recurrentis derived from B. duttoni, or that both have derived from a common ancestral strain. The results of JC Scott at al. showed that the differences in sequenced genome between those two, are as great as the differences within the species, raising the question whether these spirochetes are indeed a different species. (2) (10) Those findings were supported by S.J. Cutler et al. reporting that B. recurrentis is a subset of B. duttoni and represents a louse-adapted ecotype rather than a species (3), that has gone through a reductive evolutionary path. (2)

The optimum treatment for LBRF has not yet been fully established. A variety of drugs have been shown to be effective in removing spirochetes, such as tetracyclines, erythromycin, chloramphenicol and penicillin. But there is currently no consensus about the best suitable drug. A meta-analysis by G. Guerrier and T. Doherty comparing antibiotic regimens, including 6 RCT’s form Ethiopia found tetracycline to be significantly superior to penicillin for fever clearance time and relapse rates, while Penicillin seemed less likely to induce Jarisch-Herxheimer Reactions (JHR). Treatment regimens were most commonly single dose of 600’000 U Penicillin or 250mg Tetracycline, administered intravenously. (11) A close monitoring and optional intravenous fluids are recommended considering the danger of JHR, occurring in up to 90% of LBRF cases, (8) other reports mentioning up to 76% cases, particularly when spirochaete numbers are high. (2) General recommendations foor prophylaxis are based on hygiene, delousing and reducing of overcrowded situations. In high-risk situations, post-exposure prophylaxis using tetracycline may be considered. (8)

Several authors had considered that RF in general may be currently quite a neglected tropical disease (NTD). It has been cautioned that relapsing fever is underreported and that it is often not considered in the DD of febrile illnesses. (12) With a broad differential diagnosis ranging from Malaria to Yellow fever, it may be tricky to get a quick diagnosis. Many cases of relapsing fever present as “treatment-resistant malaria”. Diagnosis is too frequently missed through erroneous labelling of these febrile patients as malaria cases, leading to the assumption that the disease is a microbiological curiosity (2)

Taking these facts into account, we aim to collect the current knowledge about the impact of LBRF, and formulate suggestions to further studies in this field, as it may still be underreported.

# Results of Scoping searches/summary of existing literature:

Search on PROSPERO determined no planned or ongoing systematic review on this topic. Literature search about LBRF has shown rather limited information. Relapsing fever in most cases being a subtopic of Lyme Disease. Early scoping searches on MEDLINE with a focus on LBRF have provided about 94 results, with approximately 60-70% of publications relevant to the review question. Roughly a third is connected to migration. The snapshot of published evidence shows us rather few relevant studies and information about LBRF, especially limited in high level evidence studies such as RCT’s. With some authors already mentioning the suspicion that it may be a neglected disease, it gives us further reason for our study rationale.

# Study rationale:

Taking in account the results from our scoping search and the migration flow due to critical political situations in East Africa, the LBRF suggests to currently have a major impact in eastern Africa and the countries that are affected by the migration flow. Refugee camps along the way of the migration flow are ideal spots for the body louse to thrive. In favour of a better understanding of the epidemiology of this disease, it is important to know about the Pediculus humanus humanus, the vector of B. recurrentis:

Body lice are ectoparasites, of the family Pediculidae, specialized on humans. Their size is between 3 and 4 millimetres. The life cycle of the body louse consists of three stages; the nits, the nymphs and the mature form. Nits are generally easy to see on the clothing and body hair. They are oval and usually yellow to white in colour, taking up to 1–2 weeks to hatch. Nymphs are the immature louse forms and mature into adults during approximately 9–12 days. If separated from their hosts, lice die within days. (13) Body lice have a high tolerance to temperature changes, no significant differences was found by A. Gallardo et al. in the survival of body lice kept at 6ºC, 24ºC, and 31ºC for 1 or 2 days. (14) They live in symbiosis with the bacteria Candidatus Riesia, which produces the essential Vitamin B5, which the body louse can’t synthesize itself. Living on clothing and body hair, the louse feasts itself on human blood. (15) They act as vectors for the transmission of Epidemic typhus (Rickettsia prowazekii), Trench fever (Bartonella quintana) and Relapsing fever (B.recurrentis). (16) Body lice infestation can be found worldwide but generally is limited to humans who live under conditions of crowding and poor hygiene, such as: the homeless, refugees, migrants, survivors of war or natural disasters. Infestations can spread rapidly under such conditions. Body lice are spread through direct contact with an infested person or through contact with articles such as clothing, beds, bed linens, or towels that have been in contact with an infected person. (13)

In this review, we aim to raise awareness to a possibly neglected disease and finally show knowledge gaps that would suggest further studies to be conducted researching this illness. Finally, we aim to reveal what is known about these topics and what needs to be found out to improve the possibilities of treatment and prophylaxis in future.

# Review questions/objective:

Quantitative objectives to identify:

What is the current state of knowledge about the epidemiology and the geographical spread of LBRF?
What are Risk Factors associated to acquiring LBRF and for mortality?
What is the economic impact of LBRF?

More specifically:
-Collect the published literature about the chosen aspects of LBRF
-Critically assess the grade of evidence of the collected literature
-Summarize the evidence and create a critical overview with stratification by quality of report
-Identify relevant research and knowledge gaps

Further aims beyond the research question:
-LBRF being a neglected tropical disease (NTD)
-Find Risk Factors for Mortality from LBRF
-Symptoms and frequency of JHR, possibly find associations with Risk Factors
-Identify possible areas of infection along the migrants/refugee’s ways

# Inclusion criteria:

All types of studies with good evidence, such as RCTs related to LBRF will be included. Focus will be on epidemiology, economy and clinical impact. Studies including information about these topics will be considered. All types of studies with unclear grade of evidence will be furtherly assessed by two independent reviewers. Exclusion criteria is clearly poor evidence, such as newspaper articles. Date limitation will be set, probably around 1900. This may be slightly customized during the search and after discussion with the supervisor.

Based on the review questions form above, the Inclusion Criteria will be defined as:
Who/Population = All patients with LBRF
What = Impact of LBRF
How/on what = Economy, Clinic, Epidemiology
Where = Global
Grade of evidence = Good OR average - after discussion with second reviewer
Study design = RCT’s, non-randomized controlled trials, possibly any others after a check with second reviewers and grade of evidence
Date limitation = 1900 until 01/10/2017

| Screening and Selection Tool | | |
| --- | --- | --- |
| Reviewer Name: | | Date: |
| Author name/Study ID: | | Year: |
| Title: | | Journal: |
|  | Include | Exclude |
| Who/Population | Patients with LBRF | Patients with other diseases |
| What | Impact of LBRF | Not concerning LBRF |
| How/on what | Economy Clinical information Epidemiology | Not containing any Economical, Clinical or Epidemiological information |
| Where | Global (East Africa OR migrants with origins in East Africa) | - |
| Grade of evidence | Good OR average (after agreement between reviewers) | Clearly poor |
| Study design | RCT’s OR non-randomized controlled trials OR possibly any others after a check with second reviewers and grade of evidence | Newspaper articles |
| Date limitation | 01/JAN/1907 - 17/JUN/2019 | Any material before 01/JAN/1907 |

Table 1: Screening and Selection Tool

# Outcomes, intervention and phenomena of interest:

Clinical information about LBRF, furtherly specified in the data extraction list in the Appendix, are of interest to the review, as well as any economical information about costs or impact of LBRF. By conducting a wide-open search without geographical limitation, we are interested whether we will find any indicators of LBRF currently thriving in other regions of the world than only limited to East Africa. Another phenomenon of interest is the impact caused by LBRF being a rather neglected disease. Finally, any clues about possible sites of infection along the way of migrants are phenomena of interest.

# Types of studies:

Since the search strategy is rather broad and the estimated amount of studies rather low, this review will consider all types of published study designs, not only limited to RCTs. Quality assessment will be crucial before definite inclusion. In doubt, two independent reviewers will be consulted for further assessment. What types of studies eventually will be included, will become apparent after completing the search and quality assessment. Focus will be on published papers in English language. German, French and Spanish papers will be reviewed without constrictions by one of the authors, sufficiently understanding the language and using dictionaries. In doubt, further languages will be managed by outsource translation. We aim to have no language bias in selecting the studies for the review.

# Search and selection strategy:

The search strategy aims to identify all relevant published studies using the following approach:

1: Primarily, an electronic search will be conducted on CINAHL, Cochrane Library, EMBASE ovid and Elsevier, Ovid MEDLINE, PUBMED, PMC, Web of Science, Biosis, Current Contents Connect and SCOPUS electronic databases using following terms:

((Relapsing AND fever AND (Louse OR Lice OR (Pediculus AND humanus))) OR (Borrelia AND recurrentis) OR LBRF)

2: Identified material will be de-duplicated both by automatic search for duplicates by Endnote software and manual search for duplicates, following this review. Titles and abstracts of material identified via searches using the keywords above (Stage 1: Screening) will be screened and reviewed manually. Ideally, screening will be conducted by two reviewers. Alternatively, screening will be conducted by one reviewer, in doubt, a second reviewer will be consulted. Secondly, the refence lists of identified relevant articles will be manually searched for additional studies or articles. Further identified material will be again screened and reference lists will be searched.
3: Full text papers of potentially eligible articles will be obtained
4: Inclusion criteria will be applied and full text papers selected for the review. Studies that didn’t fulfil the criteria for inclusion will be excluded and their bibliographic details will be listed in an Appendix (Stage 2: Selection)
5: results will be reported using PRIMSA diagram

The bibliographic software ENDNOTE will be used for storage and processing.

# Assessment of methodological quality:

The selected full text papers (after Stage 2: Selection) will be assessed one reviewer for methodological validity prior to inclusion into the review. Any disagreements will be resolved through discussion between the reviewers, further disagreement will be intended to be resolved by a third independent reviewer.

6 key steps will be followed to assure a proper quality assessment:

1: The study designs of all the included studies will be evaluated and noted.
2: In case of a variety of designs, design-specific assessment tools will be used.
3: Appropriate quality assessment tools will be chosen
4: Quality assessment will be carried out using the right tool, carefully documenting where in the studies information relating each quality assessment question was found. Records will be kept of how the reviewer made a decision, especially if he was unable to clearly response to a question.
5: The results will be tabulated, summarized, stratified and presented in the quality assessment section if applicable.
6: It will be considered and discussed how the quality assessment results might have an impact on the conclusions and recommendations of the review. Discussion will be presented in the “Discussion” section of the review.

Finally, at the end of writing the review, it will be checked using a systematic review quality assessment tool, the PRISMA checklist.


Data extraction:
Quantitative data will be extracted and included in the review in form of comprehensive tables. The extracted data will include specific details of significance to the review objective. Details about diagnostic measures, treatment regimens and any other information of significance or interest to the review objective will be noted and documented. It is not planned to extract qualitative data. Deadline for obtaining studies in the review is set on the 17/JUN/2019.
In detail, the data extraction process will contain the following steps:
1: Any data that seems relevant for the review question will be identified and data extraction form and tables will be created. Stylistic rules of the favoured journal for publishing will be followed as far as possible at this stage.
2: The preformed data extraction form will be piloted using 3-5 of the selected studies to assess the viability of the data extraction form and possibly add or remove variables.
3: Data extraction will be conducted electronically using electronic versions of the paper. This process will be set after the quality assessment. Data will be stored in the data extraction form (Excel) using “copy and paste” to minimize data entry errors. This process will be conducted by one reviewer. Alternatively, depending on the availability of a second reviewer, a second reviewer will crosscheck the extracted data for accuracy, or the first reviewer will conduct a second full data extraction at least one week after the first data extraction to ensure proper data entry and that same results are obtained from both extractions.
4: Data extraction tables will be completed and reported in the review

# Data synthesis, discussion and context:

The data will be visually presented in summary tables and synthesized narratively in a sense of an observational analysis. As far as the available data allows, associations and conclusions will be drawn. Where we can, we will attempt to group similar data.

At this stage, the decision will be made whether the data will only be synthesized narratively or if the data is sufficient for a meta-analysis. Four aspects will be assessed whether it is appropriate to combine the results in a meta-analysis.
1: Studies should be similar in terms of the patients (inclusion criteria, patient characteristics)
2: Interventions/Exposures and Comparators should be the same
3: The same outcomes should be reported (primary or secondary, as well as time frames)
4: The results should show that the effects/impacts are generally going into the same direction (visualized by forest plot using a statistical software)

If all four criteria are sufficiently fulfilled by the data from reviewed studies, a meta-analysis will be performed – due to rather expectable lack of sufficient homogenic data, further planning in this direction isn’t appropriate now. In case of only some studies meeting all the criteria, it may be considered to perform a meta-analysis only using those studies. In this case, a sensitivity analysis will be carried out, using the remaining studies to test the robustness of the results. If all included studies meet most of the criteria, it may as well be sensible to combine them in a meta-analysis. Both cases would be carefully discussed among the reviewers and it would be clearly reported in the review, that not all criteria were met and what the implications thereof may have on results. Any decisions will be justified in the text of the review, clearly setting out the reasons of why a meta-analysis was performed or not.

As the scoping search showed a relatively limited number of published studies, we may expect limitations due to a lack of data. It is likely in this review, that we will have to deal with a variety of different study designs with different study aims, resulting in a rather big heterogeneity. Furthermore, the review question isn’t just comparing two different interventions on the outcome, we want to get a further picture of LBRF. Examining the epidemiology, the impact on economy and the clinical impact, we will most likely have to deal with a big variety of data or possibly a big lack of data. Since one of the aims of our review is to show the current state of knowledge, discovering possible knowledge gaps, this will be considered and discussed in the “discussion” and “conclusions” section.

The results section will be followed by a “discussion” section.

# Timetable:

-Deadline for obtaining studies into the review will be 17/JUN/2019.
-Screening and selecting process should be terminated around 17/OCT/2019.
-Quality assessment: roughly one hour per study should be enough for proper evaluation.
-Data extraction: roughly two hours per study would be the ideal time to carefully extract data.
-Data synthesis, discussion and conclusion will be started after quality assessment and data extraction will be terminated. Estimating three hours per study, it heavily depends on how many studies will be selected. Ideally synthesis can be started before the end of October. Roughly planning one week for synthesis, one week for discussion and conclusion, a raw version of the review might be presented to the supervisor in mid-November.

# Authors:

Pascal Kahlig, Daniel H. Paris, Andreas Neumayr

# Literaturverzeichnis

1. **Cutler, S.J.** Relapsing fever - a forgotten disease relvealed. *Journal of Applied Microbiology.* 2010, 2009, Bd. 108.

2. **S.J. Cutler, A. Abdissa, J.F. Trape.** New concepts for the old challenge of African relapsing fever borreliosis. *European Society of Clinical Microbiology and Infectious Disease.* 2009, Bd. 15.

3. **S.J. Cutler, E.M. Bonilla, R.J. Singh.** Population Structure of East African Relapsing Fever Borrelia spp. *Emerging Infectious Disease.* 2010, Bd. 16.

4. **Cutler, S.J.** Possibilities for relapsing fever reemergance. *Emerg Infect Dis.* 2006, Bd. 12.

5. **International Organization for Migration. [Online] [Zitat vom: 04. 09 2017.] https://www.iom.int/east-africa-and-horn-africa.**

**6. Houhamdi, L. and Raoult, D. Excretion of living Borrelia recurrentis in feces of infected human body lice. *J Infect Dis.* 2005, Bd. 191.**

**7. al., A.D. Bryceson el. Louse-borne Relapsing Fever. *QJ Med.* 1970, Bd. 39.**

**8. Gill, Geoffrey V. und Beeching, Nick. *Tropical medicine. Lecture notes.* Chichester : Blackwell Pub, 2009.**

**9. Tesfayohannes, T. Prevalence of body lice in elementary school students in three ethiopian towns at different altitudes. *Ethiop Med J.* 1989, Bd. 27.**

**10. al., J.C. Scott et. Typing African Relapsing Fever Spirochetes. *Emerging Infectious diseases.* 11, 2005.**

**11. G. Guerrier, T. Doherty. Comparison of antibiotic regimens for treating louse-borne relapsing fever: a meta-analysis. *Royal Society of Tropical Medicine and Hygiene.* 2011, Bd. 105.**

**12. Barbara Detrick, John L. Schmitz, Robert G. Hamilton. *Manual of molecular and clinical laboratory immunology.* Washington, DC : ASM Press, 2016 .**

**13. [Online] [Zitat vom: 19. 09 2017.] https://www.cdc.gov/parasites/lice/body/biology.html.**

**14. A. Gallardo, G. Mougabure Cueto, M. I. Picollo. Pediculus humanus capitis (head lice) and Pediculus humanus humanus (body lice): response to laboratory temperature and humidity and susceptibility to monoterpenoids. *Parasitology research.* 2009, Bd. 105.**

**15. Rademacher, Rochus. www.wissenschaft.de. [Online] 22. 06 2010. [Zitat vom: 19. 09 2017.] http://www.wissenschaft.de/home/-/journal_content/56/12054/992263/.**

**16. Bonilla, DL, et al. The biology and taxonomy of head and body lice--implications for louse-borne disease prevention. *PLOS .* 27. March 2017.**
