# Supplementary material for: Louse-borne relapsing fever—A systematic review and analysis of the literature: Part 1—Epidemiology and diagnostic aspects
Source: PLoS Negl Trop Dis. 2021 Mar 11;15(3):e0008564. doi: 10.1371/journal.pntd.0008564 (PMC7951878; doi:10.1371/journal.pntd.0008564)
Supplement: S2 Text — Used for screening and selecting eligible publications. (DOCX) [file pntd.0008564.s003.docx]

**S2 Text. Data Extraction Sheet**

| Screening and Selecting Tool – Data extraction Sheet | | | | | | |
| --- | --- | --- | --- | --- | --- | --- |
| Reviewer name: | | | | | **Date:** | |
| Author name/study ID/country: | | | | | **Year:** | |
| Title: | | | | | **Journal:** | |
| Studies excluded because: | | | | | | |
| Not about B. recurrentis | | Language | Type D evidence quality | No historical, clinical, epidemiological or economical information | | other |
|  | |  |  |  | |  |
|  | **Case report:** | | | | | |
|  | **Case series:** | | | | | |
|  | **Fever studies**: | | | | | |
|  | **Prospective fever study**: | | | | | |
|  | **Cohort study**: | | | | | |
|  | **RCT**: | | | | | |
|  | **Epidemiological study**: | | | | | |
|  | **Non-randomized CT**: | | | | | |
|  | **Other**: | | | | | |
|  | **Historic**: | | | | | |
|  | **Economics**: | | | | | |
|  | **Background/Book Sections/Informative**: | | | | | |
|  | **Mortality**: | | | | | |
|  | **Morbidity**: | | | | | |
|  | **N. of patients**: | | | | | |
|  | **Gender**: | | | | | |
|  | **Mean/Median age**: | | | | | |
|  | **Age range**: | | | | | |
|  | **Origins of patients: in A. Nr. & %**: | | | | | |
|  | **Occupation in A.Nr. & %**: | | | | | |
|  | **Status (refugee, migrant, local) in A. Nr. & %**: | | | | | |
|  | **Social (urban, rural, ref. camp) in A. Nr. & %**: | | | | | |
|  | **Way of migration**: | | | | | |
|  | **Duration of migration**: | | | | | |
|  | **Camps along the way**: | | | | | |
|  | **Other/Comments**: | | | | | |
|  | **Nr. Of Diagnosed LBRF**: | | | | | |
|  | **Country of Diagnosis**: | | | | | |
|  | **Country of Manifestiation**: | | | | | |
|  | **Laboratory confirmed A (Molecular)**: | | | | | |
|  | **Laboratory confirmed B (MALDI-TOF)**: | | | | | |
|  | **Laboratory confirmed C (Paired dynamics Serology)**: | | | | | |
|  | **Laboratory suspected D (Microscopy)**: | | | | | |
|  | **Laboratory suspected E (Serology)**: | | | | | |
|  | **Laboratory suspected F (Clinical)**: | | | | | |
|  | **Signs of ectoparasites**: | | | | | |
|  | **Random Diagnosis**: | | | | | |
|  | **Co-Diagnosis**: | | | | | |
|  | **Comments**: | | | | | |
|  | **Fever in % and A.N**: | | | | | |
|  | **Chills**: | | | | | |
|  | **Fever/Symptoms before admission (d)**: | | | | | |
|  | **Blood pressure (v.a. Hypo)**: | | | | | |
|  | **Pulse/Tachycardia in % and A.n**: | | | | | |
|  | **Headache in % and a.n.**: | | | | | |
|  | **Myalgia in % and a.n.**: | | | | | |
|  | **Arthralgia in % and a.n.**: | | | | | |
|  | **Hepatomegalia**: | | | | | |
|  | **Splenomegalia**: | | | | | |
|  | **Signs of Haemorrhage**: | | | | | |
|  | **Epistaxis in % and a.n.**: | | | | | |
|  | **Petechial rush in % and a.n.**: | | | | | |
|  | **Jaundice in % and a.n.**: | | | | | |
|  | **Nausea**: | | | | | |
|  | **Vomiting**: | | | | | |
|  | **Abdominal pain**: | | | | | |
|  | **Cough**: | | | | | |
|  | **Diarrhea**: | | | | | |
|  | **Neurological symptoms**: | | | | | |
|  | **Other symptoms and signs (jeweils in % and a.n.)**: | | | | | |
|  | **Laboratory**: | | | | | |
|  | **Thrombozytes**: | | | | | |
|  | **Number of fever relapses** (range and average): | | | | | |
|  | **Time between manifestation and 1^st^ relapse (days)**: | | | | | |
|  | **Time between 1^st^ and 2^nd^ relapse (days)**: | | | | | |
|  | **Time between 2^nd^ and 3^rd^ relapse (days**): | | | | | |
|  | **Time between 3^rd^ and 4^th^ relapse (days**): | | | | | |
|  | **Time between 4^th^ and 5^th^ relapse (days):** | | | | | |
|  | **Range Time between relapses (days**): | | | | | |
|  | **Other/Comments**: | | | | | |
|  | **Nr. of P. treated**: | | | | | |
|  | **% of P. treated**: | | | | | |
|  | **Nr. of P. untreated**: | | | | | |
|  | **% of P. untreated**: | | | | | |
|  | **Treatment regimens (drugs, dosage, duration, route of administration**): | | | | | |
|  | **Days under therapy (mean & median)**: | | | | | |
|  | **Spirochete disappearance time (h**): | | | | | |
|  | **Fever clearance time (h)**: | | | | | |
|  | **Therapy outcome**: | | | | | |
|  | **“Reaction after treatment”**: | | | | | |
|  | **Frequency of JHR**: | | | | | |
|  | **Severity of JHR**: | | | | | |
|  | **Treatment of JHR**: | | | | | |
|  | **i v. fluids before therapy.**: | | | | | |
|  | **No fluids before therapy:** | | | | | |
|  | **JHR prevention measures**: | | | | | |
|  | **Complications**: | | | | | |
|  | **Other/Comments**: | | | | | |
|  | **Mortality treated in A. Nr.**: | | | | | |
|  | **Mortality treated in %**: | | | | | |
|  | **Mortality untreated in A.Nr**.: | | | | | |
|  | **Mortality untreated in %**: | | | | | |
|  | **Cause of death**: | | | | | |
|  | **Time of death after treatment**: | | | | | |
|  | **Other/Comments**: | | | | | |
|  | **Days hospitalized (mean & median)**: | | | | | |
|  | **Hospitalized days treated**: | | | | | |
|  | **Hospitalized days untreated (or not Diagnosed)**: | | | | | |
|  | **Complications**: | | | | | |
|  | **Days in ambulant therapy (mean & median)**: | | | | | |
|  | **Follow up**: | | | | | |
|  | **Outcomes**: | | | | | |
|  | **Quality of life**: | | | | | |
|  | **Adverse events**: | | | | | |
|  | **QALY**: | | | | | |
|  | **DALY**: | | | | | |
|  | **Lost days per capita (not able to work/daily routine)**: | | | | | |
|  | **Other/Comments**: | | | | | |
|  | **Differential Diagnosis**: | | | | | |
|  | **Co-infections per patient (mean & median)**: | | | | | |
|  | **Co-infections range**: | | | | | |
|  | **Days until Diagnosis (mean & median)**: | | | | | |
|  | **Other/Comments**: | | | | | |
|  | **Insurance type**: | | | | | |
|  | **Costs for therapy**: | | | | | |
|  | **Costs per P. (mean & median)**: | | | | | |
|  | **Economical information**: | | | | | |
|  | **Other/Comments**: | | | | | |
|  | **Prevention/Prophylaxis**: | | | | | |
|  | **Interesting points/quotes/conclusions**: | | | | | |
|  | **Relevant references**: | | | | | |
|  | **Implications to possible risk factors**: | | | | | |
|  | **Comments/Other/historic**: | | | | | |
