# Supplementary figures and images for: Louse-borne relapsing fever—A systematic review and analysis of the literature: Part 1—Epidemiology and diagnostic aspects
Source: PLoS Negl Trop Dis. 2021 Mar 11;15(3):e0008564. doi: 10.1371/journal.pntd.0008564 (PMC7951878; doi:10.1371/journal.pntd.0008564)

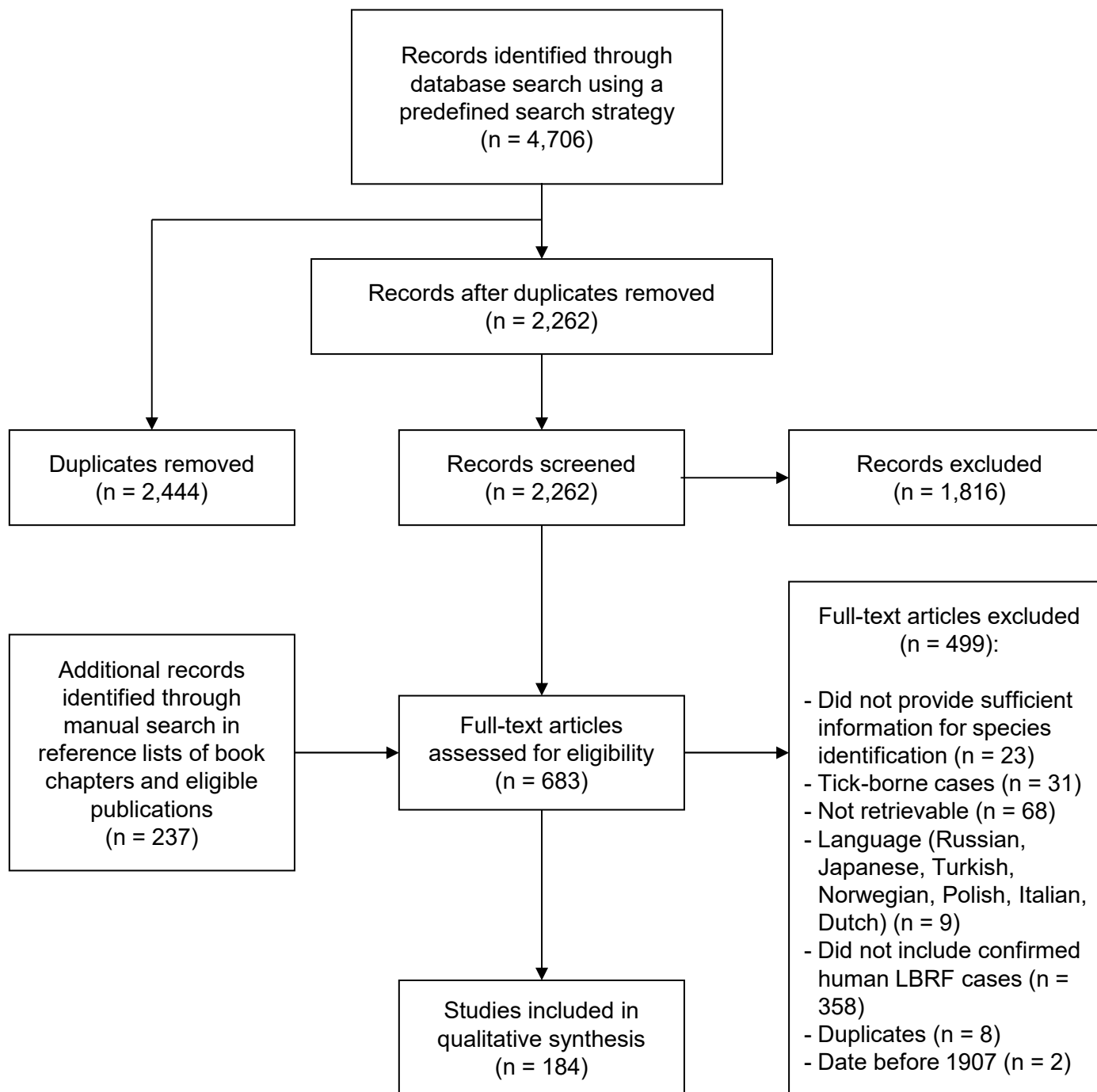

Supplement: S1 Fig — (PDF) [file pntd.0008564.s005.pdf]
